# Effectiveness of interventions to support the transition home after acute stroke: a systematic review and meta-analysis

**DOI:** 10.1186/s12913-022-08473-6

**Published:** 2022-08-28

**Authors:** Geraldine O’Callaghan, Martin Fahy, Paul Murphy, Peter Langhorne, Rose Galvin, Frances Horgan

**Affiliations:** 1 RCSI School of Physiotherapy, RCSI University of Medicine and Health Sciences, Dublin 2, DO2 YN77 Ireland; 2 Division of Population Health Sciences, RCSI University of Medicine and Health Sciences, Dublin, Ireland; 3 RCSI Library, RCSI University of Medicine and Health Sciences, Dublin, Ireland; 4 Institute of Cardiovascular and Medical Sciences, University of Glasgow, Glasgow, G31 2ER UK; 5 School of Allied Health, Faculty of Education and Health Sciences, Ageing Research Centre, Health Research Institute, University of Limerick, Limerick, V94 T9PX Ireland

**Keywords:** Stroke, Transition, Intervention effectiveness, Systematic review, Meta-analysis

## Abstract

**Background:**

Effective support interventions to manage the transition to home after stroke are still mostly unknown.

**Aim:**

The purpose of this systematic review was to investigate the effectiveness of support interventions at transition from organised stroke services to independent living at home.

**Methods:**

The Cochrane Central Register of Controlled Trials, six databases including MEDLINE and Embase, trial registries, grey literature, and Google Scholar were all searched, up to June 2021.

We included randomised controlled trials enrolling people with stroke to receive either standard care or any type of support intervention from organised stroke services to home. The primary outcome was functional status.

Two authors determined eligibility, extracted data, evaluated risk of bias (ROB2), and verified the evidence (GRADE). Where possible we performed meta-analyses using Risk Ratios (RR) or Mean Differences (MD).

**Results:**

We included 17 studies. Support interventions led to important improvements in functional status, as determined by the Barthel Index up, to 3-months (MD 7.87 points, 95%CI:6.84 to 19.16; 620 participants; five studies; I^2^ = 77%). Results showed modest but significant functional gains in the medium to long-term (6–12 month follow up, MD 2.91 points, 95%CI:0.03 to 5.81; 1207 participants; six studies; I^2^ = 84%). Certainty of evidence was low.

Support interventions may enhance quality of life for up to 3-months (MD 1.3,95% CI:0.84 to 1.76), and reduce depression (SMD -0.1,95% CI:-0.29 to − 0.05) and anxiety (MD -1.18,95% CI:-1.84 to − 0.52) at 6–12 months. Effects on further secondary outcomes are still unclear.

**Conclusions:**

Incorporating support interventions as people who have experienced a stroke transition from hospital to home can improve functional status and other outcomes. Due to study heterogeneity, the essential components of effective transition of care interventions are still unknown. Adoption of core outcome sets in stroke research would allow for greater comparison across studies. Application of a development and evaluation framework engaging stakeholders would increase understanding of priorities for stroke survivors, and inform the key components of an intervention at transition from hospital-to-home.

**Trial registration:**

CRD42021237397 - https://www.crd.york.ac.uk/prospero

**Supplementary Information:**

The online version contains supplementary material available at 10.1186/s12913-022-08473-6.

## Introduction

People recovering from acute stroke experience significant challenges in self-management of hospital-to-home transitions as they adjust to a new diagnosis, a change in health status and a realisation of ongoing care needs [1]. Many stroke survivors leave hospital with complex and on-going needs of rehabilitation and support to relearn skills and abilities; to learn new skills; to adapt to limitations caused by stroke; and to meet social, emotional and practical needs at home and in the community. Interventions such as early supported discharge (ESD), provided at the transition from hospital-to-home, reduce length of hospital stay and healthcare expenditure in stroke care [2].

The term “transition of care” is complex, challenging to define, and is often used interchangeably with other terms including care co-ordination, navigation of care and continuity of care. It encompasses both the clinical aspect of care transfer, as well as the needs of the stroke patient and their caregiver. Transition of care is defined as: “a set of actions designed to ensure the co-ordination and continuity of health care as patients transfer between different locations or different levels of care” [3]. Interventions at care transitions are acknowledged as essential to care co-ordination, impacting on quality of care and harmful incidents [4, 5].

An opportunity exists for support interventions (e.g. educational programs, individualised discharge plan), delivered when stroke survivors are transitioning from structured stroke services to their homes, to promote continuity and quality of care, enhance functional outcomes, decrease healthcare costs, and enhance user experience [6, 7]. However, there is a lack of knowledge around effective support interventions to more efficiently manage transitions for this complex health condition.

Bettger et al., 2012, considered the benefits or harms of interventions at the transition home after hospitalisation for stroke or myocardial infarction (MI) (e.g. cross-care case management, self-management tools, shared access to information, and discharge planning), and found low-to-moderate strength evidence of the effectiveness of hospital-initiated transitional care [8]. Evidence for chronic disease management care models, education, or community-based models of support for individuals with stroke or MI was inadequate [8]. An updated search in 2019, found little to add to the understanding of what components are effective at the hospital-to-home transition after stroke [9].

This systematic review and meta-analysis explores the effectiveness of support interventions at transition from structured stroke services to independent living at home on functional status and other clinical and process outcomes for stroke survivors, their families and caregivers.

### Patient and public involvement (PPI)

Patient and public involvement (PPI) was embedded in this review. Stroke survivor champions, caregivers and healthcare professionals, purposively recruited to a PPI panel, and representative of different geographic locations, genders and varied journeys along the stroke pathway, partnered with researchers. The aim of this PPI and researcher partnership was to identify, evaluate, and summarise the findings in a way that is relevant and meaningful to people impacted by stroke, and to health policy makers and practitioners.

## Methods

### Study design

This review was performed according to PRISMA standards [10] (Supplemental material, Table 2). The protocol is published on PROSPERO and in Health Research Board (HRB) Open Research [11], and the systematic review and meta-analysis was performed in accordance with this protocol. Amendments to the protocol can be found in Supplemental material, Table 1.

### Study identification

A comprehensive search of Cochrane Central Register of Controlled Trials, and six additional databases (MEDLINE, EMBASE, CINAHL, Cochrane Library, APA PsychoINFO, SCOPUS) was performed from inception to June 23rd, 2021 (Supplemental material, Table 3). We also searched a clinical trial registry (ClinicalTrials.gov), bibliographies of review papers, previous systematic reviews, grey literature, and Google Scholar. Authors of published abstracts were contacted to elicit full-text copies of studies; while authors of included studies were contacted to request study data where applicable.

### Study selection

We included randomised, controlled trials (including cluster and quasi-randomization) in adult stroke survivors, who were discharged from structured stroke services (hospital, inpatient rehabilitation, ESD) to home, and allocated to treatment with a support intervention (e.g. patient booklet, stroke passport, goal-setting, individualised discharge plan, etc.). Control groups received standard care.

We excluded ESD interventions as an evidence synthesis has been completed [2, 12]; trials published only in conference literature; or where the full-text could not be translated into English or was unavailable. We also excluded interventions where the sole focus of the support was targeting the carer.

The primary outcome was functional status, categorised as per the ‘activities’ component of the International Classification of Functioning, Disability and Health Framework, while secondary outcomes included clinical, process, and caregiver outcomes assessed in the first year of discharge. Adverse events, expected and unexpected, were examined.

### Data extraction

Two reviewers (GO’C, RG) screened titles and abstracts independently and in duplicate. Two reviewers (GO’C, FH) extracted data describing the characteristics of the included papers using standardised forms [11]. Data were presented in table form and using a transitional care framework proposed by Bettger et al., 2012 [8].

### Quality assessment

The Cochrane tool for assessing risk of bias version 2 (RoB 2) was applied to assess study quality i.e. 5 domains with risk of bias classified as “low risk of bias”, “some concerns” or “high risk of bias”2) [13]. Discrepancies between two reviewers (GO’C, FH) at each stage were resolved through discussions [11].

### Certainty of evidence

The GRADE (Grading of Recommendations, Assessment, Development and Evaluations) framework and categories (high, moderate, low, or very low) was used to determine overall certainty in the evidence [11, 14, 15].

### Meta-analysis

Meta-analysis was executed using Review Manager 5 (RevMan5) [16]. Treatment effects were determined after intervention and at follow-up intervals. Mean differences (MD) and 95% confidence intervals (CI) were pooled for continuous outcomes; standardised mean difference (SMD) and 95% CI where the scale for continuous outcomes varied; and risk ratios and 95% CI for dichotomous outcomes. Prevalence of adverse events were analysed as dichotomous variables.

Heterogeneity was determined by examining forest plot images and the I^2^ statistic. Assuming homogeneity across studies we initially used a fixed-effects (FE) model and 95% CI in meta-analysis. Where the I^2^ revealed > 50%, indicating potential clinical or methodological heterogeneity, we computed using a random effect (RE) model and 95% confidence intervals.

Sensitivity analysis was carried out to determine the impact of high risk of bias; selection bias; quasi-randomisation; missing outcomes bias; and entering assumed values on the robustness of findings for each outcome. Studies in each condition were excluded manually, and the changes in the forest plot were captured and discussed.

For residual heterogeneity, pre-planned sub-group analysis (duration of intervention; studies that recruited people with stroke and their caregivers; studies with a theoretical underpinning; studies that delivered a component specifically to the caregiver) sought to identify possible origins [17].

Where statistical pooling was unachievable, the findings are presented in table and narrative form.

**Fig. 1:**
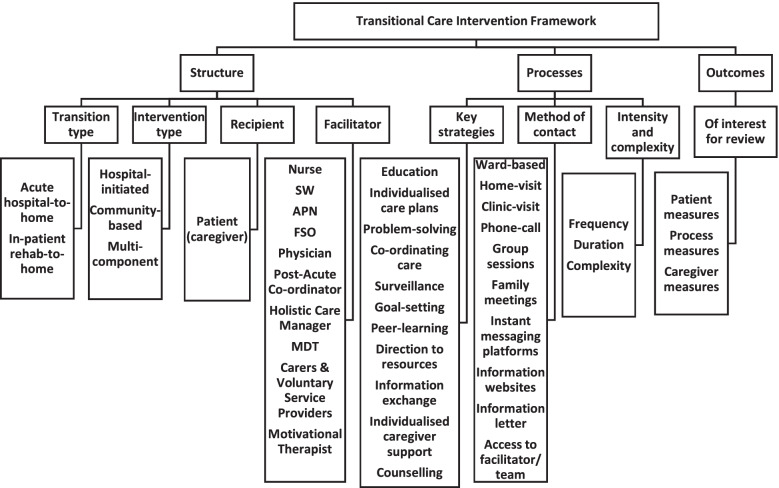
Transitional Care Intervention Framework

Using a transitional care framework (Fig. 1), proposed by Bettger et al. 2012 [8], the components of transitional care: Structure (type of transition; intervention type; recipient; facilitator); Processes (key strategies; method of contact; intensity and complexity); and Outcomes (patient, caregiver and process measures) are presented.

### Public and patient involvement (PPI)

The research question was informed by a round-table PPI consultation process with individuals who were impacted by stroke. Stroke champions (*n* = 6), caregivers (*n* = 3) and healthcare professionals (*n* = 2) worked collaboratively with the researcher to inform on the outcomes that are a priority for support interventions to target, and to interpret and discuss the findings. Discussions took place through meetings held on a video platform (Zoom), and by e-mail. Freely available interactive software tools such as mentimeter (mentimeter.com) and jamboard (jamboard.google.com) facilitated engagement, idea sharing and consensus building. The Guidance for Reporting Involvement of Patient and Public version 2 short form (GRIPP2-SF) [18] is used to report on PPI in this review (Supplemental material, Table 5).

## Results

### Results of the search

**Fig. 2:**
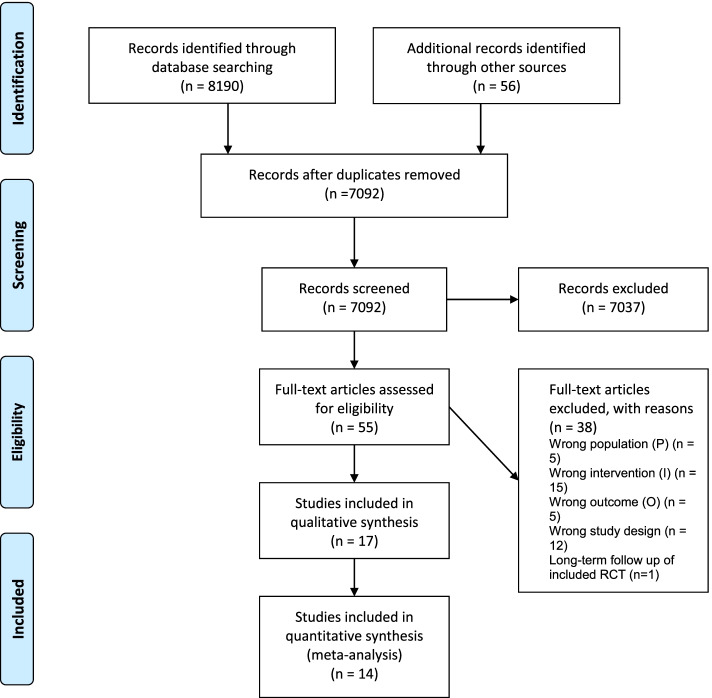
PRISMA Flow Diagram

Searches yielded a total of 8246 potentially relevant studies and 55 full-text papers were screened for eligibility. We identified a total of 17 studies eligible for inclusion (Fig. 2) with 14 studies available for meta-analysis.

### Characteristics of included studies

A total of 17 studies, from 8 geographic locations (China (n = 6 [19–24]); UK (n = 3 [25–27]); USA (n = 3 [28–30]); Australia (n = 1 [31]); Netherlands (n = 1 [32]); Thailand (n = 1 [33]); Canada (n = 1 [34]); Hong Kong (n = 1 [35])) were included in this review. One RCT tested two support intervention types (social work case management and social work case management and a website) against a control (usual care) [30].

The total number of stroke participants from included studies was 8783. Sample sizes ranged from 33 [28] to 6024 [29] participants. Overall the mean age ranged from 60 [33] to 76 [24, 26] years. The proportion of female participants ranged from 17% [28] to 63% [35]. Data for ethnicity, support system, urban/rural, and communication status were reported in some papers but not in others.

The components of included studies are summarised in Table 1; while more detailed characteristics of included studies are presented in Supplemental material, Table 5.

**Table 1:** Summary Table of Intervention

**Transition type**	Acute to home	[19–21, 23, 24, 26, 27, 29, 30, 32–34]	**Method of contact**	Inpatient	[19–22, 24–27, 29, 31, 33–35]
				Phone call	[19–24, 28–30, 32–35]
				Family meetings	[35]
	Rehab to home	[28, 31]		Home visit	[20, 21, 23–25, 27, 28, 30–35]
	Acute / Rehab to home	[25]			
				Outpatient visit	[26, 27, 29]
	Unclear	[22, 24, 35]		Group session	[19, 23, 26]
**Intervention type**	Hospital initiated; community based	[19–22, 24–27, 29, 31, 33–35]		Information website	[30]
				Information letter	[29]
	Community based	[23, 28, 30, 32]		Telephone access to facilitator / team	[24, 33, 34]
**Recipient (recruited)**	Patient	[19–24, 27–30, 34, 35]			
	Patient and caregiver/spouse	[25, 26, 31–33]		Instant messaging platform	[20, 22]
**Facilitator**	Nurse	[19, 23, 29, 32, 34, 35]			
	Social worker	[28, 30, 31]	**Length of intervention**	4 weeks	[23, 27, 33, 35]
	Multidisciplinary team	[20–22, 24, 26, 33]		6 weeks	[19, 26, 34]
				2 months	[24]
	Family support officer	[25]		3 months	[20, 21, 28–30]
	Motivational therapist	[27]		5 months	[31]
	Post-acute co-ordinator	[29]		6 months	[22, 32]
				9 months	[25]
	Physician	[29]			
**Key strategy**	Education (stroke and its management, incl. Risk factor and medication management)				[19–26, 28–35]
	Goal setting				[19, 23, 27, 30, 35]
	Problem solving				[19, 23, 27, 28, 32]
	Surveillance and ongoing support (including clinical review)				[19–22, 24, 28–30, 33–35]
	Counselling (including active listening around stroke related stress and other issues)				[25, 27, 28, 30–35]
	Individualised caregiver support				[28–30, 34]
	Bi-directional information exchange				[20, 22, 24, 26, 33, 34]
	Signposting and linking to available resources				[25, 26, 28–34]
	Individualised care plan				[20, 24, 29, 30, 33]
	Care co-ordination including onward referral				[20, 25, 28–30, 33–35]
	Peer learning				[23, 26]

#### Structure

Participants were recruited from either acute stroke (n = 11) [19–21, 23, 26, 27, 29, 30, 32–34] or inpatient rehabilitation settings (n = 2) [28, 31]. Recruitment setting was unclear in three studies [22, 24, 35], while one study recruited from both settings [25]. No participants were recruited on transition from ESD.

For all studies, the interventions were delivered to the stroke patient; and to the caregiver or spouse in 5 studies (29%) [25, 26, 31–33]. Intervention delivery was facilitated by a registered or advanced practice nurse (n = 6); multidisciplinary team (n = 6); social worker (n = 3); family support organiser (*n* = 1); advance practice provider / physician (n = 1); post-acute nurse coordinator (n = 1); or motivational therapist (n = 1).

In four studies [23, 28, 30, 32] participants were recruited pre-discharge and the intervention was solely delivered in the community setting.

#### Process

Support intervention processes were also heterogeneous in terms of content, method of contact, duration, intensity and outcomes measured. Interventions were delivered in-person (n = 17), and via telephone/letters/instant messaging platforms (n = 13) and virtually (n = 1). Eleven distinctive intervention components were used across the 17 studies, with most describing educational intervention (n = 16). Other key strategies included surveillance (n = 11); signposting (n = 9); care-coordination (n = 8); counselling (n = 9); goal setting (n = 5); problem solving (n = 5); bi-directional information exchange (n = 6); individualised care planning (n = 5); and peer learning (n = 2). Four studies also incorporated individualised caregiver support into their intervention.

Usual care was either not characterised (n = 5); was determined by primary care physician (n = 2); or varied from health education with information leaflets, to home visits, follow up calls and onward referrals.

#### Outcomes

Greater than 70 distinctive outcome measures were reported. The length of follow-up ranged from 7 days to 12 months. The most frequently reported follow-up periods were 3-months (n = 10) and 6-months (n = 7).

Theories reported as the foundation underpinning the different interventions were found in eight out of the seventeen studies [19, 21, 23, 24, 29–31, 35].

### Risk of bias in included studies

**Fig. 3:**
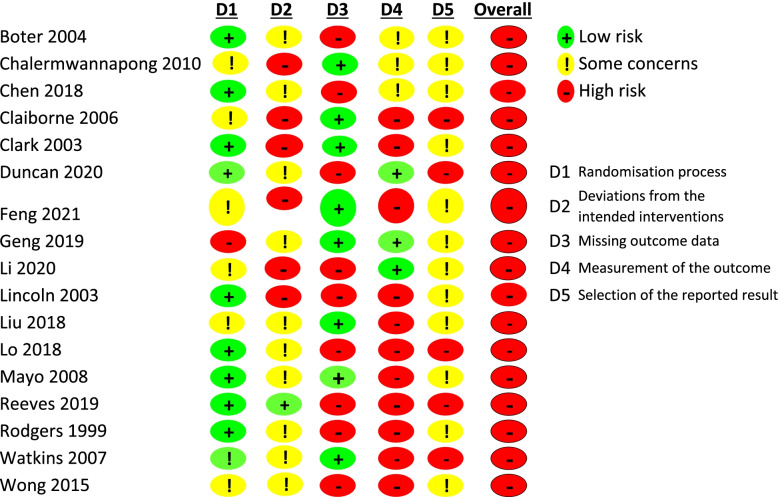
Risk of Bias of Included Studies

The risk of bias of included studies is summarised in Fig. 3, with full details of Risk of Bias in Supplemental material, Table 6. Overall methodological quality of the included studies was low, with all studies regarded as having high risk of bias.

### Effects of interventions

**Fig. 4:**
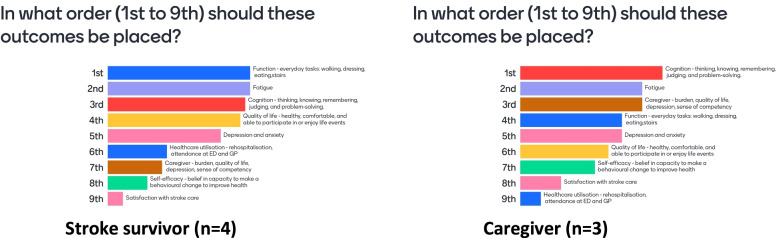
Prioritisation of Outcomes

A PPI outcomes prioritisation exercise identified the top three outcomes stroke survivors and caregivers would like impacted by a support intervention delivered at the transition from hospital to home: function, fatigue and cognition (Fig. 4). Secondary outcomes are reported according to the ranking order of stroke survivor prioritisation.

### Primary outcome

#### Functional status

Fourteen studies assessed a measure of functional status. The most frequently reported measurement of functional status was the Barthel Index (BI) [19–22, 25, 27, 31–35]. Functional status was also reported using the Modified Rankin Score (mRS) [29]; the simplified Modified Rankin Score (smRS) [30]; and the Oxford Handicap Score (OHS) [26].

**Fig. 5:**
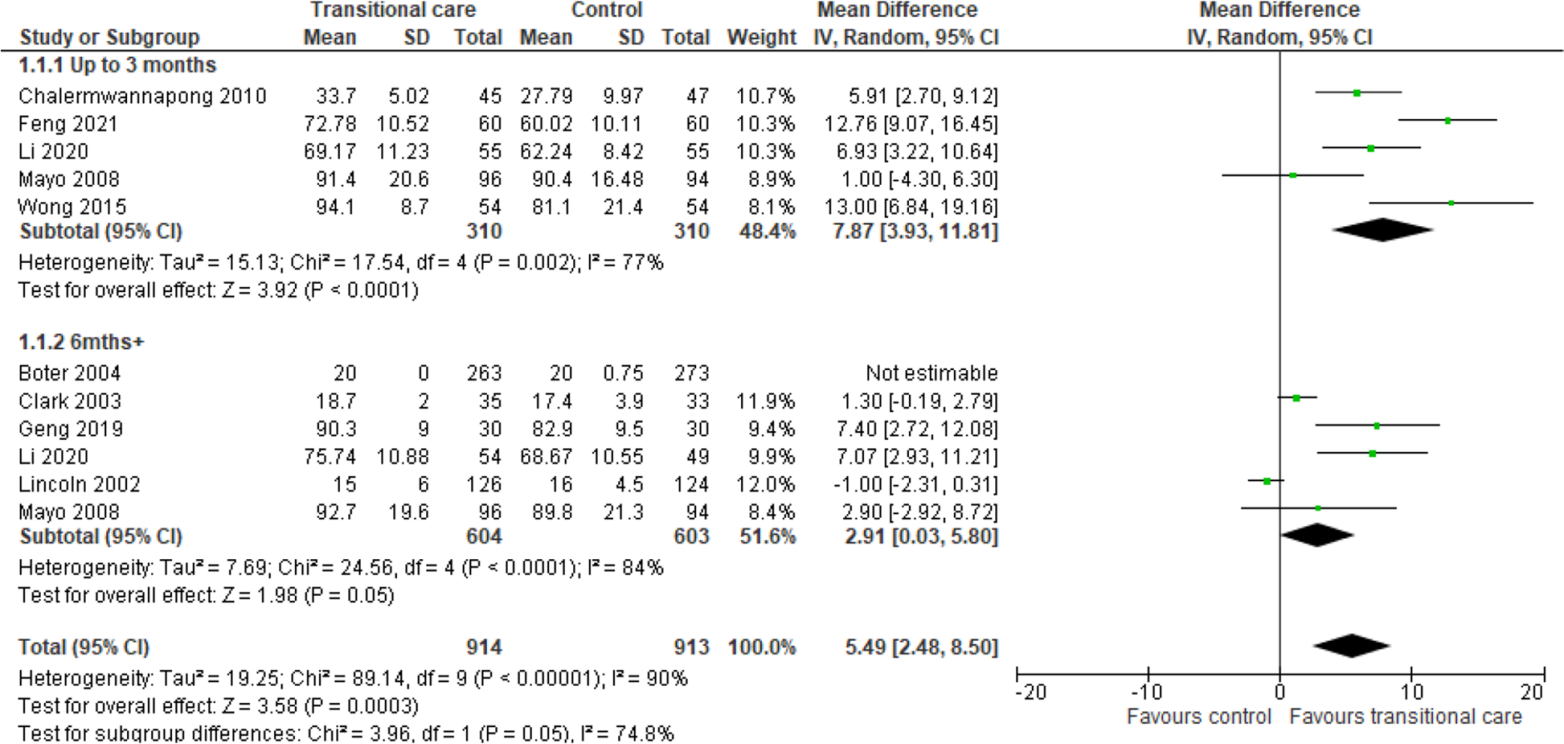
Forest Plot of Functional Status Outcome (Barthel Index): Transitional care intervention vs Control

Using the Barthel Index, five studies [20, 22, 33–35] reported effects of support intervention compared to standard control up to three months; while six studies [21, 22, 25, 31, 32, 34] reported effects in the medium-long term (> 6 months) (Fig. 5). There was an effect in favour of the intervention group up to 3-months (RE, MD 7.87, 95% CI 6.84 to 19.16, I^2^ = 77%, 620 participants; very low certainty of evidence. Studies > 6 months did not show the same degree of effect (RE, MD 2.91 points, 95% CI 0.03 to 5.81, I^2^ = 84%, 1207 participants; very low certainty of evidence. However, the minimal clinically important difference (MCID) of the Barthel Index in stroke patients is estimated to be 1.85 points, showing the effect at 6 months may still be large enough to be meaningful in the real world [36]. GRADE summary of findings can be found in Supplemental material, Table 7.

Differences in versions of BI utilised, and other pre-planned sensitivity analysis are discriminated in Supplemental material, Sensitivity Analysis I. Sensitivity analysis resulted in a larger effect in favour of the intervention group at 6–12 months (RE, MD 4.88 points, 95% CI 0.22 to 9.53, I^2^ = 82%).

### Secondary outcomes

Refer to Supplemental material for pooled results (Supplemental Files I-6 (S1-S6)) and outcome measure abbreviations (Table 5).

#### Cognition and fatigue

One cluster RCT of education, personalised care planning, and case management compared to usual care, found no effect in favour of intervention for cognition (MD -0.19, 95% CI -0.77, 0.38) or fatigue (MD 0.18, 95% CI: − 0.86, 1.22) at the end of 3-months intervention [29].

#### Quality of life

Quality of life (QoL) was measured using the SF-36 (n = 8), QLI-stroke (n = 1), Likert (n = 1), and PROMIS-10 (n = 1) tools.

At 3-months the pooled effect size for quality of life subdomains of the SF-36 [28, 34, 35] showed important between-groups differences in the Physical Component Score (PCS) (FE, MD 1.3, 95% CI 0.84 to 1.76, I^2^ = 0%), but not in the Mental Component Score (MCS) (RE, MD = 1.53, 95% CI − 1.78 to 4.85, I^2^ = 57%).

At 6–12-months [31, 34] no important between-group differences in effect was found for the SF-36 for either sub-domain – (PCS: RE, MD 0.64, 95% CI − 1.93 to 3.22, I^2^ = 65%; MCS: FE, MD 1.15, 95% CI − 1.41 to 3.72, I^2^ = 0%). Pooled results for QOL can be found in Supplemental Material, Supplemental File 3 (S3).

Sensitivity analysis found no effect in favour of the intervention for SF-36 - PCS (FE, MD 1.51, 95% CI − 1.74 to 4.77, I^2^ = 0%) at 3-months (Supplemental material, Sensitivity Analysis 2).

### Depression and anxiety

#### Depression

Eleven studies assessed impact on depression, using eight different outcomes measures (HADS-D [26, 32]; GDS [28, 31, 34]; SDS [20]); GHQ-12 [25]; GHQ-28 [27]; PHQ-2 [27]; PHQ-9 [27]; CES-D [35]).

Pooled results (Supplemental material, Supplemental File 1 (S1)) showed no important between-group difference in depression up to 3-months (RE, SMD -0.34, 95%CI -0.89, 0.12, I^2^ = 90%). At > 6 months participants receiving support intervention had lower depression scores than those receiving usual care (FE, SMD -0.17, 95%CI -0.29, − 0.05, I^2^ = 0%); effects were not sustained in sensitivity analysis (Supplemental material, Sensitivity Analysis 3).

Duncan et al. 2020 [29] reported no important between-group differences at 90-days for depression (OR 0.97, 95% CI 0.74. to 1.26).

#### Anxiety

Anxiety was measured using the HADS-A [26, 31, 32]; SAS [20]; and the NeuroQol-Anxiety [30].

There was no evidence of an effect in favour of support interventions compared to a standard control group up to 3 months (RE, SMD -0.73, 95% CI − 1.73 to 0.27, I^2^ = 95%). There was an important effect in favour of the intervention group at > 6 months (FE, MD -1.18, 95% CI − 1.84 to − 0.52, I^2^ = 0%). Pooled results for anxiety can be found in Supplemental material, Supplemental File 2 (S2). Following sensitivity analysis the results remained robust (Supplemental material, Sensitivity Analysis 4).

### Healthcare utilisation

There was no effect in favour of support interventions on hospital readmission [30, 32, 34, 35] (FE, RR 1.04, 95% CI 0.77 to 1.41, I^2^ = 6%), emergency department visits [34, 35] (RE, RR 0.43, 95% CI: 0.10 to 1.87, I^2^ = 55%), or GP visits [32, 34] (FE, RR 0.99, 95% CI 0.89 to 1.10, I^2^ = 0%). Pooled results for healthcare utilisation can be found in Supplemental material, Supplemental File 5 (S5). Following sensitivity analysis the results remain robust (Supplemental material, Sensitivity Analysis 6).

### Caregiver outcomes

#### Caregiver burden/strain

There was no evidence of effect in favour of support intervention on caregiver strain (FE, MD -0.03, 95% CI − 0.71 to 0.65, I^2^ = 0%) at > 6 months [25, 32].  Pooled results for caregiver strain can be found in Supplemental material, Supplemental File 6 (S6).

#### Caregiver depression, quality of life, sense of competency

There was little consideration of caregiver outcomes such as depression [25, 31], quality of life [26] or sense of competency [32] in included studies, and studies could not be pooled. There was no evidence in favour of support intervention in individual studies.

### Self-efficacy

Meta-analysis indicated no effect in favour of support interventions on self-efficacy i.e. an individual’s belief in their capacity to action behaviours necessary to produce specific performance goals, up to 3-months (RE, MD 0.42, 95% CI − 0.10 to 0.94, I^2^ = 90%) [20, 22, 23, 30], or between 6- and 12-months (RE, MD 0.39, 95% CI − 0.75 to 1.52, I^2^ = 90%) [20, 22]. Pooled results for self-efficacy can be found in Supplemental material, Supplemental File 4 (S4). Sensitivity analysis on studies up to 3-months showed an effect in favour of the intervention (Supplemental material, Sensitivity Analysis 5).

A motivational interviewing intervention [27] reported limited between-group differences at 90-days for self-efficacy (MD 0.8, 95% CI − 0.2 to 1.8).

### Satisfaction with stroke care

No important between-group differences were found for satisfaction with stroke care in three studies (RR 1.07, 95% CI 0.89 to 1.21) [32]; (RR 0.08, 95% CI − 0.05 to 0.20) [29]; (*p* = 0.20) [25]. By contrast, one RCT found significant between-group differences at 4 and 8-week follow-up points (*p* < 0.0001) [35].

### Adverse events

No between-group difference were found at 90 days (OR 1, 95% CI 0.83 to 1.21) for falls [29].

#### Subgroup analysis

We found no consistency in the findings across pre-planned subgroup analyses. These are presented in Supplemental material, Subgroup Analysis 1-5.

## Discussion

This systematic review found that support interventions, improved functional status and a component of quality of life in the short-term, and depression and anxiety in the medium to long-term, when compared to a control. Certainty of evidence was low. There was no evidence of effect on self-efficacy, healthcare utilisation and caregiver strain. There were little data available reporting the effect of support interventions on fatigue, cognition, satisfaction with stroke care, or caregiver outcomes.

Despite improvements in acute stroke care internationally, gaps remain in community re-integration, and in self-management after stroke [7]. Our findings suggest that multi-component support interventions improve short-term function. However, these interventions appear to have less impact on functional status 6-months after the transition period. Evidence suggests that outcomes achieved through self-management strategies are difficult to sustain, and that enhanced self-efficacy is a key facilitator in successful and sustaining effects of self-management programs [37]. This indicates that self-efficacy should be an intended outcome of self-management programs. A greater understanding of the strategies that facilitate long-term self-efficacy is required. Recent literature describes how healthcare providers and health systems need to extend beyond standard self-management strategies and to tailor self-management support to “each individual, their life context, and the realities of their illness trajectory” [38].

We found that support interventions impacted positively on anxiety and depression in long-term stroke survivors. Post-stroke depression impacts 30 to 40% of people with stroke [39]; while post-stroke anxiety is seen in 20–25% of people with stroke [40]. These neuropsychiatric disorders may have an impact on the mood and quality of life of caregivers, as well as worsen the physical and cognitive symptoms of the stroke. Evidence suggests that anxiety and depression remain highly prevalent in long-term stroke survivors [39] and, if left untreated can interfere with recovery and adversely affect functional and social outcomes. It is therefore increasingly important to explore measures to sustain benefits achieved by support interventions; and to identify the components of support that might impact on outcomes.

Two specific aspects within the included studies warrant more attention. Firstly, the diversity of outcomes reported and tools used, along with variations in length of follow-up, contributed to heterogeneity within the review and limited the generalisability of the findings. The use of a core outcome set in stroke has the potential to improve the quality and efficiency of healthcare, facilitating shared decision-making and allowing system-level comparisons. The recommended core set involves the use of patient-reported outcome measures (PROMs), which describe health status from the patient’s viewpoint [41]. An expert panel co-ordinated by International Consortium for Health Outcome Measures (ICHOM) defined a minimum set of outcomes that are a high priority to collect in stroke research. The recommended tools are the PROMIS-10, which covers multiple domains affected by stroke, supported by the mRS [41, 42]. The mRS was used in two studies, as their primary measure of function [29, 30], and the PROMIS-10 in one [30], therefore these could not contribute to our meta-analysis.

Secondly, development of trials, using an iterative consensus building approach across relevant stakeholders, and designed to fit into an existing, tenable funding mechanism follows recommendations for development of complex interventions [43]. Despite recommendations for stakeholder engagement in effectiveness trials [44], only one included study intervention design was informed by input from patients, caregivers, healthcare providers, and policymakers [29]. Duncan et al’s cluster-based trial [29] engaged multiple stakeholders to ensure patient centeredness and to optimise provider uptake in the real world. The study experienced some issues reported across other pragmatic trials i.e. challenges with intervention delivery (staff shortages, patients reluctant to attend outpatient visits), and loss to follow up. Additional research should identify hospital-level factors that are associated with higher levels of engagement and more effective implementation of interventions in pragmatic trials. Duncan et al. [29] also explored the impact of intervention on outcomes such as fatigue and cognition, highlighting the importance of these outcomes for people who have experienced a stroke and other stakeholders in the US [29], similar to UK prioritisation research [45]; and the feedback from our PPI group. Future research should consider the impact of interventions on outcomes of importance to stroke survivors and caregivers.

This review has a number of limitations. Firstly, the quality of the trials, with high risk of bias, inconsistency and imprecision, limits the certainty of evidence. Furthermore, few studies reported on outcomes such as cognition and fatigue, which may greatly influence functional performance and are important to stroke survivors. Most studies of transitional care did not include caregiver outcomes which can impact resource use and costs for the healthcare system, and there was limited reporting of adverse events.

There are a number of strengths associated with this review. The review was methodologically robust according to the PRISMA reporting guidelines. PPI and other stakeholders co-developed the research question and evaluated the outcomes that are priorities for stroke survivors and caregivers and reflected this in reporting; and guaranteed that findings were discussed in a way that considers what is meaningful and relevant to people impacted by stroke.

### Clinical implications

This review suggests that transition of care support interventions may have a short-term impact on functional status after stroke. The estimate of effects has limited certainty for chronic stroke survivors, but does extend to clinical significance [36] showing the effect at 6-months may be large enough to be meaningful in a real world context.

This review also suggests that support interventions provided at the hospital-to-home transition may have a long-term impact on those presenting with mood disorder, although we are not yet clear which intervention components have these modifying effects.

### Policy implications

The Stroke Alliance For Europe (SAFE) recommend all European countries implement frameworks for support after stroke, ensuring an integrated approach to tackling “life after stroke” issues [46]. While this review offers limited clarity on effective intervention strategies to support people as they leave hospital and go home after stroke, it directs us towards priorities for further research.

### Future research

This review highlights the need for researchers to adopt structured frameworks, such as the updated Medical Research Council (MRC) guidance [47], to inform and guide the development and evaluation of a complex intervention such as, at the transition of care from hospital to home. Development and evaluation requires stakeholder engagement and asks how the evidence supports real-world decision-making.

Following advice set out by ICHOM, future interventional research targeting the transition from hospital to home after stroke, should adopt core outcomes sets recommended for stroke, as the value of the Standard Set in clinical research will only be discernible if it is implemented and field tested [41].

### Public and patient involvement (PPI)

PPI contribution throughout a systematic review requires a level of experience, training and skill. Contributors completed an informal educational session on systematic reviews, providing them with a basic understanding of the systematic review process, and allowed them engage at select points in the review i.e. preliminary stage, design, data analysis and interpretation. This level of engagement allowed us to identify aspects that are of relevance to the intended users of the review, to pin-point future research priorities, and to inform a dissemination strategy for this research.

## Conclusion

Considering the challenges faced by stroke survivors and caregivers during the transition from acute stroke services to their homes, a better understanding of what interventions are effective in supporting this transition is urgently needed. We found that multi-component interventions appear to positively impact on function, at least in the short-term; and patient anxiety and depression in the longer-term. The effect on other clinical, process, and caregiver outcomes remains uncertain. Implementing a support intervention that is effective and sustains outcomes for stroke survivors will require the application of a development and evaluation framework that engages all stakeholders and delves into the nuances of a complex intervention to increase understanding.

## Supplementary Information

**Additional file 1: Supplemental Tables** (Table 1- Table 7). **Supplemental Files** (S1 - S6). **Supplemental Sensitivity Analysis** (1 - 6). **Supplemental Subgroup Analysis** (1 – 5).

## Data Availability

The datasets generated and/or analysed during the current study are available in the Zenodo repository, https://zenodo.org/search?page=1&size=20&q=6779371
