# Supplementary material for: Effectiveness of interventions to support the transition home after acute stroke: a systematic review and meta-analysis
Source: BMC Health Serv Res. 2022 Aug 28;22:1095. doi: 10.1186/s12913-022-08473-6 (PMC9420257; doi:10.1186/s12913-022-08473-6)
Supplement: Supplementary file 1 — Additional file 1: Supplemental Tables (Table 1- Table 7). Supplemental Files (S1 - S6). Supplemental Sensitivity Analysis (1 - 6). Supplemental Subgroup Analysis (1 – 5). [file 12913_2022_8473_MOESM1_ESM.docx]

**SUPPLEMENTAL MATERIAL**

**Effectiveness of interventions to support the transition home after acute stroke: a systematic review and meta-analysis**

**Authors and Affiliations:**

**Geraldine O’Callaghan**^1^, Martin Fahy^2^, Paul Murphy^3^, Peter Langhorne^4^, Rose Galvin^5^, Frances Horgan^1^

^1^ School of Physiotherapy, RCSI University of Medicine and Health Sciences, Dublin 2, DO2 YN77, Ireland
^2^ Division of Population Health Sciences, RCSI University of Medicine and Health Sciences, Dublin, Ireland
^3^ RCSI Library, RCSI University of Medicine and Health Sciences, Dublin, Ireland
^4^ Institute of Cardiovascular and Medical Sciences, University of Glasgow, Glasgow, G31 2ER, UK
^5^ School of Allied Health, Faculty of Education and Health Sciences, Ageing Research Centre, Health Research Institute, University of Limerick, Limerick, V94 T9PX, Ireland

Corresponding author: Geraldine O’Callaghan. School of Physiotherapy, RCSI University of Medicine and Health Sciences, Dublin 2, DO2 YN77, Ireland.

Email: gocallaghan@rcsi.com

Telephone: +353 (0)85 1217559

**Supplemental material included**:

Supplemental Tables (Table 1- Table 7)

Supplemental Files (S1 - S6)

Supplemental Sensitivity Analysis (1 - 6)

Supplemental Subgroup Analysis (I – 5)

References (as per main document)

**Table 1: Protocol amendments**

|  | Specified in protocol | Amendment | Justification |
| --- | --- | --- | --- |
| Population | Studies that included Transient Ischaemic Attack (TIA) or subarachnoid haemorrhage were accepted if > 80% of the participants have had an ischaemic stroke | Studies that included participants with a confirmed diagnosis of stroke. | Many studies did not define type of stroke. |

**Table 2: PRISMA Checklist**

| **Section and Topic** | **Item #** | **Checklist item** | **Location where item is reported** |
| --- | --- | --- | --- |
| **TITLE** | | |  |
| Title | 1 | Identify the report as a systematic review. | 1 |
| **ABSTRACT** | | |  |
| Abstract | 2 | See the PRISMA 2020 for Abstracts checklist. | 2/3 |
| **INTRODUCTION** | | |  |
| Rationale | 3 | Describe the rationale for the review in the context of existing knowledge. | 4/5 |
| Objectives | 4 | Provide an explicit statement of the objective(s) or question(s) the review addresses. | 5 |
| **METHODS** | | |  |
| Eligibility criteria | 5 | Specify the inclusion and exclusion criteria for the review and how studies were grouped for the syntheses. | 6  &  Protocol |
| Information sources | 6 | Specify all databases, registers, websites, organisations, reference lists and other sources searched or consulted to identify studies. Specify the date when each source was last searched or consulted. | 6 |
| Search strategy | 7 | Present the full search strategies for all databases, registers and websites, including any filters and limits used. | Supplemental material  (page 6/7) |
| Selection process | 8 | Specify the methods used to decide whether a study met the inclusion criteria of the review, including how many reviewers screened each record and each report retrieved, whether they worked independently, and if applicable, details of automation tools used in the process. | 7  &  Protocol |
| Data collection process | 9 | Specify the methods used to collect data from reports, including how many reviewers collected data from each report, whether they worked independently, any processes for obtaining or confirming data from study investigators, and if applicable, details of automation tools used in the process. | 7  &  Protocol |
| Data items | 10a | List and define all outcomes for which data were sought. Specify whether all results that were compatible with each outcome domain in each study were sought (e.g. for all measures, time points, analyses), and if not, the methods used to decide which results to collect. | 7  &  Protocol |
|  | 10b | List and define all other variables for which data were sought (e.g. participant and intervention characteristics, funding sources). Describe any assumptions made about any missing or unclear information. | 7  &  Protocol |
| Study risk of bias assessment | 11 | Specify the methods used to assess risk of bias in the included studies, including details of the tool(s) used, how many reviewers assessed each study and whether they worked independently, and if applicable, details of automation tools used in the process. | 7  &  Protocol |
| Effect measures | 12 | Specify for each outcome the effect measure(s) (e.g. risk ratio, mean difference) used in the synthesis or presentation of results. | 8  &  Protocol |
| Synthesis methods | 13a | Describe the processes used to decide which studies were eligible for each synthesis (e.g. tabulating the study intervention characteristics and comparing against the planned groups for each synthesis (item #5)). | 8  &  Protocol |
|  | 13b | Describe any methods required to prepare the data for presentation or synthesis, such as handling of missing summary statistics, or data conversions. | 8  &  Protocol |
|  | 13c | Describe any methods used to tabulate or visually display results of individual studies and syntheses. | 8  &  Protocol |
|  | 13d | Describe any methods used to synthesize results and provide a rationale for the choice(s). If meta-analysis was performed, describe the model(s), method(s) to identify the presence and extent of statistical heterogeneity, and software package(s) used. | 8  &  Protocol |
|  | 13e | Describe any methods used to explore possible causes of heterogeneity among study results (e.g. subgroup analysis, meta-regression). | 8  &  Protocol |
|  | 13f | Describe any sensitivity analyses conducted to assess robustness of the synthesized results. | 8  &  Protocol |
| Reporting bias assessment | 14 | Describe any methods used to assess risk of bias due to missing results in a synthesis (arising from reporting biases). | 7  &  Protocol |
| Certainty assessment | 15 | Describe any methods used to assess certainty (or confidence) in the body of evidence for an outcome. | 7-8  &  Protocol |
| **RESULTS** | | |  |
| Study selection | 16a | Describe the results of the search and selection process, from the number of records identified in the search to the number of studies included in the review, ideally using a flow diagram. | 10 |
|  | 16b | Cite studies that might appear to meet the inclusion criteria, but which were excluded, and explain why they were excluded. | N/A |
| Study characteristics | 17 | Cite each included study and present its characteristics. | 10-13  & Supplemental material |
| Risk of bias in studies | 18 | Present assessments of risk of bias for each included study. | 13  & Supplemental material |
| Results of individual studies | 19 | For all outcomes, present, for each study: (a) summary statistics for each group (where appropriate) and (b) an effect estimate and its precision (e.g. confidence/credible interval), ideally using structured tables or plots. | 14-18  &  Supplemental material |
| Results of syntheses | 20a | For each synthesis, briefly summarise the characteristics and risk of bias among contributing studies. | 14-18  &  Supplemental material |
|  | 20b | Present results of all statistical syntheses conducted. If meta-analysis was done, present for each the summary estimate and its precision (e.g. confidence/credible interval) and measures of statistical heterogeneity. If comparing groups, describe the direction of the effect. | 14-18  &  Supplemental material |
|  | 20c | Present results of all investigations of possible causes of heterogeneity among study results. | 14-19  &  Supplemental material |
|  | 20d | Present results of all sensitivity analyses conducted to assess the robustness of the synthesized results. | 14-19  &  Supplemental material |
| Reporting biases | 21 | Present assessments of risk of bias due to missing results (arising from reporting biases) for each synthesis assessed. | Supplemental material |
| Certainty of evidence | 22 | Present assessments of certainty (or confidence) in the body of evidence for each outcome assessed. | Supplemental material |
| **DISCUSSION** | | |  |
| Discussion | 23a | Provide a general interpretation of the results in the context of other evidence. | 19-21 |
|  | 23b | Discuss any limitations of the evidence included in the review. | 21-22 |
|  | 23c | Discuss any limitations of the review processes used. | 21-22 |
|  | 23d | Discuss implications of the results for practice, policy, and future research. | 22-23 |
| **OTHER INFORMATION** | | |  |
| Registration and protocol | 24a | Provide registration information for the review, including register name and registration number, or state that the review was not registered. | 3 |
|  | 24b | Indicate where the review protocol can be accessed, or state that a protocol was not prepared. | 6 |
|  | 24c | Describe and explain any amendments to information provided at registration or in the protocol. | Supplemental material |
| Support | 25 | Describe sources of financial or non-financial support for the review, and the role of the funders or sponsors in the review. | 24 |
| Competing interests | 26 | Declare any competing interests of review authors. | 24 |
| Availability of data, code and other materials | 27 | Report which of the following are publicly available and where they can be found: template data collection forms; data extracted from included studies; data used for all analyses; analytic code; any other materials used in the review. | Supplemental material |

**Table 3: Search Strategy for Ovid Medline, COCHRANE LIBRARY and CENTRAL REGISTRY OF CLINICAL TRIALS on Wiley, EMBASE on Elsevier.com, CINAHL on Ebscohost, APA PsychINFO on Ebscohost and SCOPUS on Elsevier.com**

|  | **Ovid MEDLINE(R)** |
| --- | --- |
| 1 | Stroke.mp. OR exp Stroke/ OR (Post stroke).mp. OR poststroke.mp. OR  Stroke rehabilitation.mp. OR exp Stroke Rehabilitation/ OR Cerebro-vascular accident.mp. OR (Cerebro vascular).mp. OR CVA.mp. OR exp cerebrovascular disease/ OR (cerebrovascular adj1 disease).mp. |
| 2 | subarachnoid hemorrhage.mp. or Subarachnoid Hemorrhage/ |
| 3 | 1 NOT 2 |
| 4 | (Continuity adj3 patient adj3 care).mp. OR exp Continuity of Patient Care/ OR (patient adj2 discharge).mp. or exp Patient Discharge/ OR (discharge adj1 plan*).mp. |
| 5 | (Transition* adj2 care).mp. OR exp Transitional Care/ OR  (Patient adj2 transition*).mp. OR (Patient adj2 handoff).mp. OR (Patient adj2 transfer).mp. OR exp Patient Transfer/ |
| 6 | Exp Patient Navigation/ OR (Patient adj2 Navigation).mp. OR *Case Management/ OR (Posthospital OR Post hospital OR Post discharge).mp. |
| 7 | 4 OR 5 OR 6 |
| 9 | Exp Home Care Services/ OR (home adj3 care).tw. OR Exp Home Care Services, Hospital-Based/ OR Exp Home Nursing/ OR Exp Primary Health Care/ OR (primary adj2 care).mp. OR (domiciliary adj 1 care).mp. OR (Community adj2 based adj2 support$) OR Exp Community Health Services/ OR (community adj1 care).mp. |
| 10 | 3 AND 7 AND 9 |

|  | **COCHRANE LIBRARY and CENTRAL REGISTRY OF CLINICAL TRIALS on Wiley** |
| --- | --- |
| 1 | Mesh Descriptor: [stroke] explode all trees OR MeSH descriptor: [Cerebrovascular Disorders] explode all trees OR Mesh Descriptor: [Stroke Rehabilitation] explode all trees OR stroke:ti,ab OR poststroke:ti,ab |
| 2 | subarachnoid NEAR/1 hemorrhage:ti,ab,kw |
| 3 | #1 NOT #2 |
| 4 | Patient NEAR/1 care:ti,ab,kw OR patient NEAR/2 discharge:ti,ab,kw OR discharge NEAR/1 plan:ti,ab,kw OR Transition NEAR/2 care:ti,ab,kw OR transitional NEAR/1 care OR Patient NEAR/1 transition:ti,ab,kw OR patient NEAR/1 handoff:ti,ab,kw OR Patient NEAR/1 transfer:ti,ab,kw |
| 5 | Mesh Descriptor:[Patient Discharge] explode all trees |
| 6 | #4 OR #5 |
| 7 | Mesh Descriptor: [Primary Health Care] explode all trees |
| 8 | Home NEAR/1 care:ti,ab,kw OR Domiciliary NEAR/1 care:ti,ab,kw |
| 9 | #7 OR #8 |
|  | #9 = Cochrane Systematic Reviews |
|  | #9 = Central Registry of Clinical Trials |

|  | **EMBASE on Elsevier.com** |
| --- | --- |
| 1 | stroke:ti,ab,kw OR Stroke/exp OR ‘Post stroke’:ti,ab,kw OR poststroke:ti,ab,kw OR ‘Stroke rehabilitation’:ti,ab,kw OR ‘acute ischemic stroke’/exp OR ‘cerebrovascular accident’/exp OR ‘Cerebrovascular accident’:ti,ab,kw OR CVA:ti,ab,kw OR ‘cerebrovascular disease’:ti,ab,kw |
| 2 | ‘subarachnoid hemorrhage’:ti,ab OR ‘Subarachnoid Hemorrhage’/exp |
| 3 | #1 NOT #2 |
| 4 | ‘Continuity patient care’:ti,ab,kw OR ‘patient discharge’:ti,ab,kw OR ‘hospital discharge’/exp OR discharge:ti,ab,kw OR ‘discharge plan*’:ti,ab,kw |
| 5 | ‘Transition* care’:ti,ab,kw OR ‘Transitional Care’/exp OR ‘Patient transition*’:ti,ab,kw OR ‘Patient handoff’:ti,ab,kw OR ‘Patient transfer’:ti,ab,kw |
| 6 | ‘Patient Navigation’:ti,ab,kw OR ‘Case Management’:ti,ab,kw OR (Posthospital OR ‘Post hospital’):ti,ab,kw |
| 7 | 4 OR 5 OR 6 |
| 9 | ‘Home Care’/exp OR ‘home care’:ti,ab,kw OR ‘Home Nursing’:ti,ab,kw OR ‘Primary Health Care’/exp OR ‘primary care’:ti,ab,kw OR ‘domiciliary care’:ti,ab,kw OR ‘Community based support$’:ti,ab,kw OR ‘community care’:ti,ab,de,kw |
| 10 | 3 AND 7 AND 9 |

|  | **CINAHL on Ebscohost** |
| --- | --- |
| 1 | (MH “Stroke+”) OR (MH “Cerebral Hemorrhage+”) OR TI stroke OR AB stroke OR "stroke rehabilitation" OR TX cerebrovascular N1 accident$ OR cerebral N1 hemorrhage |
| 2 | subarachnoid N1 hemorrhage |
| 3 | S1 NOT S2 |
| 4 | (MH "Continuity of Patient Care+") OR (MH "Patient Discharge") OR "patient discharge" OR (MH "Transfer, Discharge") OR (MH "Discharge Planning") |
| 5 | (MH "Transitional Care") OR "transitional care" OR “Patient transition* OR (Patient handoff” OR “Patient transfer” OR “patient navigation” |
| 7 | 4 OR 5 |
| 8 | (MH "Home Health Care") OR "home care" OR “domiciliary care” OR (MM "Primary Health Care") OR (primary N2 care) OR (MH "Community Health Nursing+") |
| 9 | 3 AND 7 AND 8 |

|  | **APA PsychINFO on Ebscohost** |
| --- | --- |
| 1 | TX stroke OR TX ‘Cerebrovascular Accidents’ |
| 2 | TX ‘subarachnoid hemorrhage’ |
| 3 | S1 NOT S2 |
| 4 | TX continuity N3 care OR MM "Continuum of Care" OR TX patient N2 discharge OR DE "Hospital Discharge" OR DE "Discharge Planning" OR TX (Transition* N2 care) OR TX Patient* N2 transition OR TX 'Patient handoff' OR TX 'Patient transfer' OR TX Patient N2 navigation OR TX post N1 discharge |
| 5 | TX home N1 care OR MM "Home Care" OR TX domiciliarly N1 care OR MM "Primary Health Care" OR TI Primary N3 care OR AB Primary N3 care |
| 6 | 3 AND 4 AND 5 |

|  | **SCOPUS on Elsevier.com** |
| --- | --- |
| 1 | TITLE-ABS-KEY ( ( “stroke” ) OR ( “poststroke” ) OR ( "cerebrovascular accident" ) OR ( "Stroke rehabilitation" ) OR ( "Cerebrovascular accident" ) ) |
| 2 | TITLE-ABS-KEY ( "subarachnoid hemorrhage" ) |
| 3 | #1 AND NOT #2 |
| 4 | TITLE-ABS-KEY(“continuity of care” OR “patient discharge” OR “hospital discharge” OR "discharge plan" OR "discharge planing" OR "post discharge” OR “Transitional care” OR “Patient transition” OR “patient handoff” OR “Patient transfer” OR “patient navigation”) |
| 5 | TITLE-ABS-KEY(“home care” OR “Home Nursing” OR “Primary Health Care” OR “primary care” OR “domiciliary care”) |
| 6 | 3 AND 4 AND 5 |

**Table 4:** GRIPP2 short form

| Section and topic | Item | Reported on page No |
| --- | --- | --- |
| 1: Aim | Report the aim of PPI in the study | 5 |
| 2: Methods | Provide a clear description of the methods used for PPI in the study | 9 |
| 3: Study results | Outcomes—Report the results of PPI in the study, including both positive and negative outcomes | 14 |
| 4: Discussion and conclusions | Outcomes—Comment on the extent to which PPI influenced the study overall. Describe positive and negative effects | 22-23  &  Supplemental material |
| 5: Reflections/critical perspective | Comment critically on the study, reflecting on the things that went well and those that did not, so others can learn from this experience | Supplemental material |

PPI=Patient and Public Involvement

Discussion / Reflections.

PPI contributors were provided with an informal educational session on systematic reviews, with the aim of providing them with a basic understanding of the systematic review process. Despite this educational session, the researchers felt that the PPI contributors did not have the relevant experience, training and skills to be involved throughout the review, but rather at select points i.e. preliminary stage, design, data analysis and interpretation, and dissemination.

By engaging with PPI and other stakeholders we co-developed the research question; evaluated the outcomes that are priorities for stroke survivors and caregivers and reflected this in reporting; and guaranteed that findings were discussed in a way that considers what is relevant and meaningful to people affected by stroke.

In response to PPI feedback we reported not only on clinical (e.g. functional status, quality of life, cognition etc) and process (e.g. healthcare utilisation) outcomes, but also considered outcomes of importance to caregivers’ such as psychological well-being and quality of life. PPI contributors offered insights into findings and identified aspects for discussion that would be of relevance to the intended users of the review.

This level of engagement allowed us to identify future research priorities, and further collaboration will inform a dissemination strategy.

**Table 5: Characteristics of Included studies**

| **Author; Design**  **(Country;**  **Funding)** | **Number of Participants; Age in years (Mean/Median (SD/IQR)); Gender (Female); Severity of stroke; Recruitment setting; Urban /rural; Support system; Ethnicity; Communication status** | **Intervention**  **(Theory)** | **Intervention Characteristics** | | | | **Control Group or other interventions** | **Time to follow up** | **Outcome Measures** | **Outcome findings** |
| --- | --- | --- | --- | --- | --- | --- | --- | --- | --- | --- |
|  |  |  | **Strategies** | **Facilitator**  **(level of experience)** | **Methods Used** | **Intensity/ Duration** |  |  |  |  |
| **Boter 2004^1^;**  **RCT**  **(Netherlands; Netherlands Heart Foundation, Netherlands Organization for Health Research and Development, University Medical Center Utrecht.)** | N=536 stroke pts (& caregivers)  IG=263, age median and IQR: 66, 52-76  CG=273, age median and IQR: 63, 51-74  Gender (Female):  IG:51%; CG:52%  Severity of stroke (RS):  IG: 0(18%), 1(35%), 2(32%), 3(16%)  CG (%): 0(16%), 1(32%), 2(36%), 3(16%)  Recruitment setting: Acute  Urban/Rural: NR  Support system (Live alone; can identify carer):  IG: 30%; 80%;  CG: 26%; 84%  Ethnicity: NR  Communication status: Excl. if not Dutch speaking | Outreach Nursing Support Program | Education, problem solving, reassurance and signposting | Nurse  (experienced and trained stroke nurses) | Recruited in SU  Post-discharge  PC’s and HV to Ax risk factors, consequences of, and unmet needs after stroke (checklist); Ed. via interaction and brochures; and support based on needs, with problem-solving approach and onward referral to PCP as necessary. | Intervention lasted 6 months    3 x PC’s at weeks 1-4, 4-8 & 18-24 weeks    HV 10-14 weeks | Standard Care (no details) | 6 months post discharge | Satisfaction with stroke care (SADC-19); Depression (HADS); Readmission; ADL (BI); Independence (MRS); Use of HC services; Use of secondary prevention drugs;  Caregiver: strain (CSI), Sense of competency (SCQ); Discrepancies in social support (SSL-D) | Significant between group differences found for emotional role domain in SF-36, use of rehab services and anxiety scores. |
| **Chalerm-wannapong et al. 2010^2^;**  **RCT**  **(Thailand; Thailand Nursing Council)** | N=92 stroke pts (& caregivers) recruited; 67 completed.  IG=33, age mean and SD: 60.15 +/- 10.23;  CG= 34, age mean and SD: 60.24 +/- 11.13  Gender (Female):  IG:52%; CG:32%  Severity of stroke (disability -BI):  IG: mod(21%), severe(76%), very severe (3%); CG: mod(18%), severe(79%), very severe (3%)  Recruitment setting: Acute  Urban/Rural: NR  Support system (spouse/child):  IG: 1000%; CG: 100%  Ethnicity: NR  Communication status: Able to communicate in Thai language | Informational transitional care program to foster self-management | Individualised care plan, education, signposting, counselling, care coordination, surveillance and programme adjustment | Investigator (nurse specialist) and MDT (nurses, doctors and other HCP) | Pre-discharge:  Baseline needs Ax;  Ed. and skills training (stroke  knowledge; home modification; medication; stress management, mobility/positioning; social support; ADL; exercise / physical activity; addressing specific individual needs); Provision of community DC and resource summaries, and an educational booklet and video.  Post-discharge: PCs; HVs and access to investigator number to monitor, address concerns and adjust programme | Intervention lasted for 4 weeks after DC  Pre-discharge  45-60 minutes daily  Post-discharge  2 x HV’s: at 24-48 hours, and within 2 weeks of DC;  Telephone access to investigator;  2 x F/U PC | Usual care  (provided by nursing and other HCP to include info. and general nursing care; ward round by physician; other rehab as need identified by physician) | 12 weeks | Functional ability (MBI);  QOL (Ferrans and Powers’ QLI-Stroke Version) | The study found that the functional ability and QOL of SS in the IG was significantly higher than in CG. |
| **Chen et al. 2018^3^; RCT (China; National Natural Science Fund of China)** | N=144 stroke pts  IG=72, age mean and SD: 65.92 +/- 12.8  CG=72, age mean and SD: 64.78 +/- 9.87  Gender (Female):  IG:28%; CG:25%  Severity of stroke (MRS):  IG: median 2 (0,3)  CG: median 2 (0,3)  Recruitment setting: Acute  Urban/Rural:  Urban  Support system (Live with family):  IG: 99%; CG: 100%  Ethnicity: NR  Communication status: must be able to communicate | Patient-centred self-management empowerment intervention (PCSMEI)  (Guided by Health Empowerment Theory – empowerment through collaboration to facilitate self-management) | Education, goal setting, problem solving, surveillance and positive reinforcement. | Nurse  (no detail) | Pre-discharge:  Baseline needs Ax, stroke knowledge and goals; individual advice and instruction in problem-solving, and self-health monitoring skills; group sessions to build on knowledge and skills (provision of DVD and group experience sharing); pre-DC Ax and instruction on rehab and self-management goal setting).  Post-discharge: Ax of self-management skills and behaviours (assessing pt’s performance; identifying barriers or problems and teaching problem-solving skills; identifying goal accomplishment and providing positive reinforcements and empowerment) | Intervention lasted 6 weeks  Pre-discharge:  1x Pt. centred needs, knowledge and goals Ax;  5 x daily individual 20 min self-management ed. and skills sessions (week 1); 1 x small group session; and 1 x DC instruction  Post-discharge:  4 x 20-30 min weekly PC F/U | Conventional Nursing Care  (health ed., and post-DC medical F/U. CG received the same number of PC’s -general social chatting, in order to balance the psychological effects of professional contact in the IG) | On DC, 1 month and 3 months post DC | Self-efficacy (SSEQ); Functional performance (BI); Rehospitalisation (pt. records) | The study found that the rehospitalisation rate in IG was lower than in CG, and showed clinical significance at both time points. |
| **Claiborne 2006^4^;**  **RCT** (**USA; Unclear)** | N=33 stroke pts recruited, 28 completed.  IG=16, age mean and SE: 70 +/- 13.9  CG=12, age mean and SE: 65 +/- 11.99  Gender (Female):  IG:56%  CG:17%  Severity of stroke: NR  Recruitment Setting: Rehab  Urban/Rural:  NR  Support system (Married):  IG: 81%; CG: 75%  Ethnicity (White):  IG:75%; CG:100%  Communication status: excluded if severe cognitive deficits or language impairment | Care  Co-ordination Model | Education, problem solving, signposting, care coordination, surveillance and counselling. Specific caregiver support as required. | SW  (no detail) | Recruited as inpt  Post-discharge  HV and PC for Biopsychosocial Ax; ed. and appropriate referrals; Advocacy in relation to additional service needs (across health, public and community services); Review and support of caregiver needs; Monitor pt. progress | Intervention lasted 3 months    1 x HV within 1-2 weeks of DC    1 x Weekly F/U PC (20 mins-1hour) | FU as determined by PCP | 3 months post DC | QOL (SF-36);  Depression (GDS); Adherence to medication; Service Needs Ax | The study found a significant effect on mental QOL, depression, and adherence to self-care practices, but not on physical QOL.  Service needs were omitted in analysis |
| **Clark 2003^5^; RCT**  **(Australia; Australian Rotary Health Research Fund and the Flinders Medical Centre Foundation)** | N=68 stroke pts  (& spouses) recruited; 62 completed  IG=32, age mean and SD: 73.3 +/- 8.5; CG=30, age mean and SD: 71.2 +/- 8.8  Gender (Female):  IG:41%, CG:37%  Severity of stroke: NR  Recruitment Setting: Rehab  Urban/Rural:  Urban  Support system (Married): IG: 100%; CG: 100%  Ethnicity: NR  Communication status: excl. if severe cognitive or language impairment, or poor command of English | Supporting pt. and family through education and counselling    (guided by family systems theory) | Education, counselling and signposting | SW  (sound knowledge of stroke; worked independent of team) | At discharge  General stroke and resource info. pack provided  Post-discharge  HVs to reinforce info., offer counselling around stroke related stresses, and advise re service access | Intervention lasted 5 months    3 x HV x 1 hr at 3 weeks, 2 months and 5 months after DC | Standard care (no details) | 6 months post DC | Functional status (BI); Social recovery (AAP); Health status (SF-36);  Self-efficacy (mastery scale) Family functioning (FAD); Depression (GDS); Anxiety (HADS-A)  Caregiver QOL (SF-36 -PCS, MCS) | The study found the intervention group to have better family functioning and social recovery, but no significant differences in other outcomes. |
| **Duncan et al. 2020^6^;**  **Cluster RCT**  **(USA; Patient-Centered Outcomes Research Institute Project Program Award)** | N= 6024 stroke pts recruited; 5882 completed:  IG = 2689; age, mean and SD: 68 +/- 13.8  CG = 3193; age, mean and SD: 66.3 +/- 13.9  Gender (Female):  IG:48.3%; CG: 51.9%  Severity of stroke (NIHSS; median, (IQR): IG: 1 (0-3)  CG: 1 (0-3)  Recruitment setting: Acute  Geographical area (Urban):  IG: 60.2%  CG: 80.5%  Support system (Married): IG: 100%; CG: 100%  Ethnicity (White):  IG:79.1%  CG: 67.2%  Communication status: Aphasia:  IG: 21.6%, CG: 24.5%, must speak English/Spanish. | Comprehensive Post-Acute Stroke Services (COMPASS) Transitional Care (TC) & individualised pt. electronic CP  (guided by RE-AIM framework) | Needs assessment with individualised care plan, care coordination, education, signposting, and surveillance to facilitate self-management. Specific caregiver needs Ax. and care plan as required. | Post-acute coordinator & an Advanced practice provider or Physician  (Stroke trained. Also received 2 day study training, performance reports; peer support; access to educational support) | Pre-discharge  Brochure with risk factor info. study details and timelines, and clinic apt.  Post discharge  F/U call (medication reconciliation;  monitor stroke symptoms; falls; receipt of home health, out-pt. services, and other supports; schedule F/U apts with PCP and COMPASS providers); Clinic visits for needs Ax (pt. / proxy); personalised CP (health, lifestyle, behavioural); and referrals and communication with community providers | Intervention lasted 80 days  Telephone F/U within 2 days of DC  Clinic visit between days 7 & 14  Reminder letters at 30, 60, and 80 days (study purpose, available resources, and contact info. for study staff)  Telephone F/U at 30 and 60 days to evaluate adherence to the COMPASS-CP | Usual Care  (not characterised due to variability in transitional care in hospitals. Pts in both arms received a study brochure and info. on BP control and were advised to track their BP) | 90 days after hospital DC | Functional status (SIS-16)  PROMS;  Self-reported disability (MRS); Medication adherence; Depression (PHQ-2); Cognition (MoCA); Self-rated health (Likert scale); Fatigue (PROMIS -fatigue); Care-satisfaction (CAHPS);  Mortality; Self-reported BP;  Falls; Physical activity (steps walked per day). | The study found a low level of intervention fidelity, with no outcome significantly influenced by the intervention. |
| **Feng et al. 2021^7^; RCT (China; None)** | N=120 stroke pts  IG = 60; age, mean and SD: 70.52 +/- 5.73  CG = 60; age, mean and SD: 70.27 +/- 5.41  Gender (Female):  IG:45%, CG: 48%  Severity of stroke: NR  Recruitment setting: Acute  Urban/Rural:  Urban  Support system (Family member):  IG: 100%; CG: 100%  Ethnicity: NR  Communication status: excluded if cognitive  Impairment | Hospital-community integrated service model (HCISM) | Education, care coordination, bi-directional hospital-community collaboration, individualised care plan, bi-directional patient-provider knowledge and information exchange, surveillance and programme adjustment. | Head nurse led MDT including neurologist; rehab therapists; specialist nurses; and community doctors and nursing staff  (no detail) | On admission: Individualised Ax and CP  Pre-discharge: Comprehensive Ax and summary to community team; organising HV and F/U calls  Post-discharge: PC’s and HVs to monitor and adjust programme; A pt.-community info. platform was utilised; & relay of Ax findings from community to hospital staff. | Intervention lasted 3 months  Pre-discharge:  Unclear  Post-discharge:  PC x 2 per month  HV x 1 per month  Unlimited  “We-Chat” bidirectional info. sharing and problem solving | Routine Care (Pre-DC home rehab & medication  guidance.  Monthly post-DC PC F/U). | 3 months | Self-care ability (MBI); Compliance Behaviour (change in proportion of medications prescribed); Self efficacy (GSES); Mood (SAS & SDS) | The study found all outcomes improved significantly in the IG compared to the CG |
| **Geng et al. 2019^8^;**  **Quasi-randomisation**  **(China; Ministry of Education)** | N=60 stroke pts  IG = 30; age, mean and SD: 67.6 +/- 4.3  CG = 30; age, mean and SD: 68.9 +/- 4.4  Gender (Female):  IG:33%;CG: 47%  Severity of stroke: MRS between 2 & 4  Recruitment Setting: Acute  Urban/Rural:  Urban  Support system (Married, spouse as primary caregiver):  IG: 93.3%; 53.3% CG: 93.3% ; 60%  Ethnicity: NR  Communication status: must be cognitively competent (C-MMSE >20) and able to communicate | Transitional care, based on Integrated Behavioural Theory  (guided by Health Behaviour Theory) | Education, Surveillance and ongoing support. Focus on shared accountability across providers and organisations. | Community nurse-led MDT including neurologist, neurological nurse practitioner, rehab counsellor, psychologist, and two community transitional care nurses  (no detail) | Pre-discharge:  Health ed. (face-face & booklet) to pt. and caregiver  Post-discharge:  Needs Ax, and HV’s and PC’s to monitor health behavioural implementation; HV and PC’s by MDT as required to address needs. | Intervention lasted 3 months  Pre-discharge  1 x 30 min ed. session  Post-discharge  1 x Weekly HV (at w/end)  1 x Weekly F/U PC (Tue-thus)  As required HV & F/U PC’s by MDT | Routine care (pre-DC bedside health ed. and telephone FU 1 week after DC, and physician visit at the hospital out-pt. clinic after DC) | 1, 3 and 6 months after hospital DC | Health Behaviours (MC-HPLP 11); Stroke Knowledge (Stroke knowledge Q); Metabolic Indicators (BP, BMI, Cholesterol); ADL (MBI); QOL ( SF-36); Stroke recurrence (interview and medical records) | The study found significant improvements in the IG, for metabolic indicators; ADLs; some QOL domains (physical functioning; general health; vitality and mental health); health behaviours; and stroke knowledge. |
| **Li et al., 2020^9^; RCT (China; Unclear)** | N=110 stroke pts recruited; 103 completed.  IG = 54; age, mean and SD: 68.56 +/- 6.629  CG = 49; age, mean and SD: 68.24 +/- 6.591  Gender (Female)  IG:42%; CG:37%  Severity of stroke: NR  Recruitment setting: Unclear  Urban/Rural:  Urban  Support system: NR  Ethnicity: NR  Communication status: NR | Transitional care via **cloud** “BIHUYIHU” follow up services | Education, surveillance and bi-directional info. exchange. | MDT Including postgrad nursing students and supervisor, brain branch doctors and nurses, physical therapist, psychology consultant  (no detail) | Pre-discharge:  Training to pt. & caregiver in “BIHUYIHU”  Post-discharge:  Bi-directional Info. exchange via push notification and uploads (Pts upload their feedback on info., and upload daily rehab. photos (reminders sent)); Individual consultations and  PC F/U to monitor and adjust programme | Intervention lasted 6 months  Pre-discharge  Unclear  Post-discharge  Every 3 days:  Bi-directional info. exchange  Daily upload of rehab photos  PC F/U at 1, 3 and 6 months  No detail on Individual consultations | Routine care and telephone F/U (knowledge manual and instruction on lifestyle, diet, medication, recovery exercise, self-inspection; PC F/U at 1, 3 and 6 months after DC | 1, 3 and 6 months after intervention | Self-efficacy (GSES)  Functional ability (MBI) | The study found that participants in the IG had significant improvements in self-efficacy and functional ability, compared to those in the CG. |
| **Lincoln et al. 2003^10^; RCT (UK; Stroke Association)** | N=250 stroke pts (& caregivers)  IG=126, age mean and SD: 69 +/- 11.4  CG=124, age mean and SD: 70.2 +/- 9.6  Gender (Female):  IG:47%; CG: 49%  Severity of stroke (median BI): IG: 10 (5-16), CG: 10 (6-15)  Recruitment Setting: Acute & Rehab  Urban/Rural:  Rural  Support system: Caregivers involved in research  Ethnicity: NR  Communication status (Dysphasic): IG: 38%, CG: 42% | Transitional care via Stroke Family Support Organiser ((FSO) | Education, care-coordination, counselling and signposting | Stroke Family Support Organiser (FSO)  (no detail) | Pre-discharge:  FSO provided info. re: supports and stroke to pt. and caregiver, and identified info. needs; attended case conferences; liaised with rehab team and caregivers around DC.  Post-discharge:  HV by FSO to discuss problems, offer info and emotional support, and to direct to appropriate services. | Intervention provided for up to 9 months post recruitment.  Pre-discharge:  1 x contact as in-pt  Post-discharge: HV (As determined by FSO, or by the needs and requests of pt./caregiver Frequency unclear). | Standard care (no description of usual care provided) | 4 & 9 months | Personal care (BI);  Stroke survivor and caregiver: General Health-mood (GHQ-12); Activities of daily living (NEADL),  Knowledge about stroke accessing community services, Satisfaction with FSO;  CSI | The study found no sig. diff. between groups for mood, independence in personal care or ADL’s.  Pt’s and caregivers in IG were sig. more satisfied with info. received, and knowledge about stroke, risk factors, practical help, community services and emotional support. |
| **Liu 2018^11^; RCT (China; Henan Province Medical and Academic Technological Leaders Overseas Training Program, and the Zhengzhou University School of Nursing “Emerging Nursing Discipline” Priority Program)** | N=40 stroke pts  IG = 20; age, mean and SD: 76.32 +/- 4.56  CG = 20; age, mean and SD: 74.12 +/- 3.12  Gender (Female):  IG:45%; CG:35%  Severity of stroke: NR  Recruitment Setting: Unclear  Urban/Rural:  Urban (NR)  Support system: NR  Ethnicity: NR  Communication status: NR | Transitional care intervention  (guided by Naylor transitional Care model) | Individualised care plan, education, surveillance and bi-directional information exchange (btw carer and team) | Nurse-led MDT including physicians, nurses, rehab therapists; pts and their caregivers  (The lead nurse was head nurse of the neurology ward and had professional knowledge and skills in stroke care) | At Discharge: Individualised functional and cognitive Ax followed by comprehensive rehab and educational CP to pt. & caregiver  Post-discharge:  F/U PC & HV to monitor for problems and compliance, and adjust CP  Caregivers were active members of team, implementing care updating team on patient needs | Intervention lasted 2 months  Daily F/U PCs x 1 month  HVs X 2^nd^ month (no detail provided) | Routine care (instructions at DC: functional exercise, daily care, dietary guidance, psychological care, and medication guidance) | I, 3 & 6 months after intervention | Functional exercise compliance (functional exercise compliance questionnaire)  Health status (SF-36) | The study found sig. diff. between groups for functional exercise compliance (1&3 months); some domains of health status (1 & 3 Months); and only 1 domain of each outcome at 6 months. Intervention effect decreased over time |
| **Lo et al. 2018^12^; RCT (China; Unclear)** | N=128 stroke pts (IG = 64;  CG = 64).  Overall: Mean age and SD: 67.46 +/- 11.95  Gender (Female): 41%  Severity of stroke: NR  Recruitment Setting: Acute  Urban/Rural:  Urban  Support system: NR  Ethnicity: NR  Communication status : excluded if spoke language other than Cantonese, and had limited comprehension, and receptive aphasia | Stroke self-management programme (SESSMP)  (guided by Bandura theory of self-efficacy and outcome expectation) | Education, goal setting, problem solving and peer learning | Nurse  (experienced in stroke care and self- management) | Recruited in SU  Post-discharge:  HV; PC’s; group sessions and DVD provision facilitating goal setting and development of action plans to help stroke survivors manage stroke successfully | Intervention lasted 4 weeks.  HV (week 1)  2 x 2 hour group sessions (week 2&3)  3 x F/U PC (week 4) | Usual care (e.g. health talks) | 8 weeks after randomisation | Self-efficacy (SSEQ); Outcome expectation (Stroke Self-Management Outcome Expectation Scale); and Self-management behaviours (Stroke Self-Management Behaviours Performance Scale) | The study found the IG to have significantly improved in al outcomes, both in intention-to- treat and per-protocol analysis. |
| **Mayo et al. 2008^13^; RCT (Canada; Medical Research Council of Canada (now Canadian Institute of Health Research))** | N=190 stroke pts  IG=96, age mean and SD: 70 +/- 14.5  CG=94, age mean and SD: 72 +/- 12.95  Gender (Female):  IG:33%; CG:45%  Severity of stroke (CNSS-mean and SE):  IG: 8.9 (0.24)  CG: 9.4 (0.20)  Recruitment setting: Acute  Urban/Rural:  Urban  Support system (Lives alone):  IG: 23%  CG: 23%  Ethnicity: NR  Communication status: NR | Transitional care via Nurse Case Manager (CM) | Care coordination, education, signposting, counselling, bi-directional information exchange, surveillance and ongoing support. Specific caregiver support. | Nurse  (extensive experience with geriatric nursing, including stroke) | At-discharge:  Provide DC summary to pt’s PCP (or physician at local community health centre (CLSC)) and arrange F/U apt.  Post-discharge:  24-hour telephone access to nurse    F/U PC & HV interventions to include surveillance; info. exchange; medication management; health system guidance; active listening; family support; teaching; and risk identification. | Intervention lasted 6 weeks  F/U PC’s or HV’s determined by needs basis (average 4.8 HV’s lasting 40-90 mins; 7.8 PC’s lasting 5-20 mins) | Usual care  (pt. and family were instructed to make an apt. with their PCP or at their CLSC (provided with a list of these centres). | 6 weeks after intervention  6-months post-stroke | Function  (PCS of SF-36,  MCS of SF-36; EQ-5D; PBSI  RNLI; BI; Depression ( GDS); Gait speed; TUG;  Utilisation of health services | The study found no difference between groups for primary, secondary or health care utilisation outcomes. |
| **Reeves et al. 2019^14^; 3-group RCT (USA; Patient-Centered Outcomes Research Institute (PCORI) Award)** | N= 265 stroke pts  IG (SWCM) = 88; age, mean and SD: 67.5 +/- 13  IG (SWCM + MISTT) = 90; age, mean and SD: 66.2 +/- 12.7  CG = 87; age, mean and SD: 65.2 +/- 13.9  Gender (Female): IG (SWCM): 49%  IG (SWCM + MISTT): 48%  CG: 55%  Severity of stroke (NIHSS-mean (SD)): IG (SWCM): 4.9(5.2)  IG (SWCM + MISTT): 5.5(4.4)  CG: 4.9(4.9)    Recruitment  Setting: Acute  Urban/Rural:  Urban  Support system (Caregiver participation):  IG (SWCM): 67%  IG (SWCM + MISTT): 65%  CG: 60%  Ethnicity (white): IG (SWCM): 76%  IG (SWCM + MISTT): 77%  CG: 83%  Communication status: Excl. if has cognitive/ communication deficits, and has no proxy | Social Work Case Management (SWCM) +/_ Michigan Stroke Transitions Trial (MISTT) Website  (guided by the principles of Social Work Care Model integrated with 3 components of Chronic Care Model (CCM) | Needs Ax. and individualised CP, goal setting, education, care coordination, counselling, signposting, surveillance. Specific caregiver support.  For Group 2 (SWCM + MISTT - a patient- orientated information and support website) | SW  (masters-level trained) | Recruited in SU  Post-discharge  SWCM:  Baseline biopsychosocial Ax; HV, PC, email, and text message interactions and activities (dictated by the pt and caregiver needs and preferences), and referrals to services; Close out case summary.  MITSS website:  Ax, access to, and training in use of website at initial HV; embedded in subsequent HVs and f/u calls. | 60 day intervention period (could extend to 90 days)  By day 7 HV for biopsychosocial Ax  Details from protocol:  At least 2 HVs (day 1-7, day 30);  Weekly FU PC’s (determined by needs on a case-by-case basis)  Between day 60 & day 90 complete close out case summary | Usual care (standard post-DC instructions, services, and recommendations: medication lists, ed. materials, follow-up instructions, and referrals to medical apts, and post-acute services) | Days 7 & 90 | PROMIS-10; Knowledge and self-efficacy (PAM); Depression (PHQ-9); QOL (Neuro-QOL anxiety); sMRS  ADL & iADLS  Hospital readmissions; recurrent stroke/TIA; Time away from home. | The study found (at 90 days) that SWCM with access to online info. produced significantly more gains in pt. reported physical health (PROMIS-10 GPH, ADL/IADL, s- MRS) compared with usual care or case management alone. No significant difference between groups for mental health (PHQ-9, PROMIS-10 GMH, Neuro-QOL anxiety) or self-efficacy. |
| **Rogers et al. 1999^15^;**  **RCT (UK; Northern and Yorkshire Regional Health Authority National Health Service Research and Development Directorate)** | N=204 stroke pts (& caregivers)  IG= 121, median age:74 (36-94)  CG= 83, median age:76 (36-95)  Gender (Female):  IG:51%; CG:54%  Severity of stroke (median BI): IG: 11 (0-20), CG: 9 (0-20)  Recruitment Setting: Acute  Urban/Rural:  Urban  Support system: 100% caregiver participation  Ethnicity: NR  Communication status: Excl. if unable to complete interview / no proxy | Stroke Education Programme (SEP) | Education, peer support, signposting, bi-directional information exchange | MDT including Nursing, PT, OT, SLT, clinical psychology, SW, district nursing; local carers centre; and Stroke Association Information Officer.  (no detail) | Pre- & post-discharge:  Group educational sessions with MDT to improve knowledge of stroke and stroke prevention; services available; and to ask questions | Intervention lasted 6 weeks  Pre-discharge  1 x 1-hour group educational session  Post-discharge:  6 x 1-hour weekly educational sessions (as outpt in the day hospital) | Conventional stroke unit care (info. leaflet about the stroke service (admission); directed to Stroke Association Leaflet; and access to telephone “hot-line” run by stroke unit (discharge)). | 6months | Perceived health Status (SF-36); Knowledge of stroke; Satisfaction with services; Emotional outcome (HADS); Disability; Handicap (NEADL and OHS)  Caregiver: Emotional outcome (GHQ-12); Perceived health status (SF-36) | The study found a low level of intervention fidelity, but also found significantly improved pt. and caregiver stroke knowledge and satisfaction. However,  these improvements had no impact on perceived health status, or emotional / functional outcomes |
| **Watkins et al.2007^16^; RCT**  **(UK; NHS Executive, Department of Health North West, and Royal Liverpool and Broadgreen University Hospitals NHS Trust)**  **&**  **Watkins et al.2011^17^; RCT;**  **UK** (*Long term follow up) | N=411  stroke survivors  IG=204, age, median and IQR: 70, 61-78  CG=207, age, median and IQR: 70, 61-77  Gender (Female):  IG:51%; CG: 54%  Severity of stroke (median BI): IG: 16 (9-20), CG: 16 (9-20)  Recruitment setting: Acute  Urban/Rural: NR  Support system: NR  Ethnicity: NR  Communication status: Excluded if severe cognitive and/or communication problems | Motivational Interviewing | Problem solving, goal setting, active listening | Motivational therapist  (4 days training by a specialists, 10 practice sessions, supervision by a psychologist, engaged in reflective practice) | Pre or post-discharge  Individual sessions of motivational interviewing to identify and address concerns and perceived barriers after stroke; Goal  setting using a problem solving approach. | Intervention lasted 4 weeks    4 x Motivational Interviews, 1 per week (30-60 mins) | Usual Care (MDT input, DC planning, and a Stroke Review Clinic apt. at 1, 3, and 6 months post-stroke) | 3 and 12 months post stroke | Mood (GHQ-28);  Depression (Yale); Function (BI, *NEADL); Self-efficacy (SEQ); | The study found a significant effect of MI on mood and protection against depression, but not significant impact on function or self-efficacy.  This positive impact of MI on mood was sustained at 12 months, as was the risk of mortality. |
| **Wong and Yeung, 2015^18^;**  **RCT (Hong Kong; University Research Grants Council (RP0S) of Hong Kong and the Alice Ho Miu Ling Nethersole Charity Foundation Project Grant (5-ZH64))** | N=108 stroke pts  IG=54, age mean and SD: 67.5 +/- 11.6  CG=54, age mean and SD: 71.5 +/- 11.6  Gender (Female):  IG:63%  CG:63%  Severity of stroke: NIHSS ≥ 4 & <16  Recruitment Setting: Unclear  Urban/Rural:  Rural  Support system (Married):  IG: 53.7%; CG: 68.5%  Ethnicity: NR  Communication status: Excluded if cognitive and communication deficits present | Transitional care program  (guided by Omaha framework) | Surveillance; education, counselling and care coordination. | Nurse HCM    (nurses with experience in stroke care and in the community setting; attended 3 day training workshop) | Pre-discharge:  Family meeting,  Post-discharge:  HV’s and PCs to provide participants with info. on stroke care; instruction on how to perform self-care; guidance on overcoming barriers; demonstrating physical exercises; social support and guidance on secondary stroke prevention, building resilience, self-monitoring, recovery and outcomes | Intervention lasted 4 weeks  Pre-discharge: Entrance family meeting  Post discharge:  4 x weekly HV’s (days 2-4 of each week)  PC F/U  (day 7 of each week)  Week 4 (HV and exit family meeting) | Routine Care (hospital-based Ax and physical training programme offered, as appropriate, within 3 weeks of hospital DC. | At weeks 4 & 8 post discharge | QOL (SF-36 &  WHO-QOL-SRPB); Pt. satisfaction (PSQ-HK); Functional performance (MBI); Depression (CES-D); Healthcare utilisation (hospital info. systems) | The study found between group differences in QOL, satisfaction, functional performance and depression. There was a sig. diff. in A&E visits, and reduced incidence of readmission (not sig. diff). |
| Note. All transitions in care were from hospital (acute/inpatient rehabilitation) to home.  APN = advanced practice nurse; apt. = appointment; Ax= assessment; CG = The Control Group; CM = case manager; CP = care plan; DC = discharge; ed. = education; FSO = family support organizer; F/U = follow-up; HCM = Healthcare manager; HCM = Holistic Care Manager; HCP = healthcare provider; IG = The Intervention Group; Info= information; Inpt = inpatient; MDT = Multi-disciplinary team; OT = occupational therapy; PC = phonecall; PCP = primary care provider; PT = physical therapy; pt. = patient; pt. ed. = patient education; QOL = quality of life; RCT = randomized controlled trial; SD = standard deviation; SE = standard error; SLT = speech and language therapy; SU = Stroke Unit; SW = social work; TIA = transient ischemic attack; rehab. = rehabilitation; & = and  ADL, IADL = Activities of Daily Living / Instrumental Activities of Daily Living; BI = Barthel Index; BP = blood pressure; BSFC CAHPS survey = Consumer Assessment of Healthcare Providers and Systems; CES-D = Center for Epidemiological Studies-Depression; CNSS = Canadian Neurological Stroke Scale; CSI = Caregiver Strain Index; EQ-5D = EuroQol 5 dimension; FAD = Family Assessment Device; FAT = Frenchay Arm Test (upper limb function); Ferrans and Powers QOL Index=Ferrans and Powers QOL Index; GES = Global Effectiveness Score; GDS = Geriatric Depression Scale; GHQ= General Health Questionnaire; GSES = General Self-Efficacy Scale; HADS = Hospital Anxiety and Depression Scale; HADS-A = Hospital Anxiety and Depression Scale – Anxiety; MBI = Modified Barthel Index; MC-HPLP-11 = Health-Promoting Lifestyle Profile II; MoCA = Montreal Cognitive Assessment; MRS = Modified Rankin Scale; NEADL Scale = Nottingham Extended Activities of Daily Living Scale; Neuro-QOL-anxiety = Neuro Quality of Life - anxiety subdomain; NIHSS = NIH Stroke Scale/Score; OHS = Oxford Handicap Scale; PAM = Patient Activation Measure; PBSI = Performance Based Stroke Index; PHQ-Patient Health Questionnaire ; PROMIS-10 = Patient Reported Outcome Measure Information System –10; PROMIS-Fatigue = Patient Reported Outcome Measure Information System –Fatigue; PSQ-HK = Patient Satisfaction Questionnaire Hong Kong; RS = Rakin Score; RNLI = Reintegration to Normal Living Index; SADC-19 = Satisfaction with Stroke Care questionnaire; SDS = Self-Rating Depression Scale; SAS = Self-Rating Anxiety Scale; SF-36 = Short Form – 36; SF-36 (MCS) = Short Form-36 (Mental Component Score); SF-36 (PCS) = Short Form-36 (Physical Component Score); SCQ = Sense of Competence Questionnaire; sMRS = Simplified Modified Rankin Scale; SSL-D = Social Support List-Discrepancies; SIS-16 = Stroke Impact Scale-16; SSEQ = Stroke Self-Efficacy Questionnaire; MMSE = Mini-Mental State Examination; TUG = Timed Up and Go Test; WHO-QOL-SRPB = World Health Organisation Quality of Life -Spirituality, Religiousness and Personal Beliefs); QLI = Quality of Life Index. | | | | | | | | | | |

**Table 6: Risk of Bias of Included Studies**

Overall methodological quality of the included studies was low, with all studies regarded as having high risk of bias. While sixteen studies were randomized with inclusion/exclusion criteria and appropriate allocation concealment, six studies did not report the randomization or allocation concealment methods in detail^2^ ^2, 4, 7, 9, 11, 18^, and one study, of quasi-randomisation design, was rated as high risk of bias^8^. Nine studies (53%)^1, 3, 6, 9, 10, 12, 14, 15, 18^ had missing outcomes data, while six studies (35%)^2, 4, 5, 7, 9, 10^ did not describe intention to treat analysis. Selective outcome reporting bias was found in five studies ^6, 12, 14, 16, 18^, and only four studies published pre-trial protocols ^6, 12, 14, 16^. The most common reasons for lower quality included the absence of double-blind procedure and non-blinding of outcome assessors, likely associated with the nature of the particular intervention, and the use of patient-reported outcomes measures (PROMs) in many studies.

| **Study ID** | **Outcome** | **D1** | **D2** | **D3** | **D4** | **D5** | **Overall** |  |  |  |  |  |
| --- | --- | --- | --- | --- | --- | --- | --- | --- | --- | --- | --- | --- |
| Boter 2004 | Anxiety_HADS-A |  |  |  |  |  |  |  |  | Low risk |  |  |
| Boter 2004 | Carer Burden_CBI |  |  |  |  |  |  |  |  | Some concerns |  |  |
| Boter 2004 | Depression_HADS-D |  |  |  |  |  |  |  |  | High risk |  |  |
| Boter 2004 | Functional status_BI |  |  |  |  |  |  |  |  |  |  |  |
| Boter 2004 | Functional status_RS |  |  |  |  |  |  |  | D1 | Randomisation process |  |  |
| Boter 2004 | Quality of Life_SF36 |  |  |  |  |  |  |  | D2 | Deviations from the intended interventions |  |  |
| Boter 2004 | Readmission |  |  |  |  |  |  |  | D3 | Missing outcome data |  |  |
| Boter 2004 | Satisfaction with stroke care_SASC-19 |  |  |  |  |  |  |  | D4 | Measurement of the outcome |  |  |
| Boter 2004 | Sense of competence_SCQ |  |  |  |  |  |  |  | D5 | Selection of the reported result |  |  |
| Boter 2004 | Use of GP |  |  |  |  |  |  |  |  |  |  |  |
| Boter 2004 | Use of HC services_Rehabilitation Day care |  |  |  |  |  |  |  |  |  |  |  |
| Boter 2004 | Use of HC services_Therapy |  |  |  |  |  |  |  |  |  |  |  |
| Chalermwannapong 2010 | Quality of life_QLI-stroke version |  |  |  |  |  |  |  |  |  |  |  |
| Charlemwannapong 2010 | Functional status_MBI |  |  |  |  |  |  |  |  |  |  |  |
| Chen 2018 | Functional status_BI |  |  |  |  |  |  |  |  |  |  |  |
| Chen 2018 | Readmission |  |  |  |  |  |  |  |  |  |  |  |
| Chen 2018 | Self-efficacy_SSEQ |  |  |  |  |  |  |  |  |  |  |  |
| Claiborne | QOL_SF-36 |  |  |  |  |  |  |  |  |  |  |  |
| Claiborne 2006 | Depression_GDS |  |  |  |  |  |  |  |  |  |  |  |
| Clark 2003 | Anxiety_HADS-A |  |  |  |  |  |  |  |  |  |  |  |
| Clark 2003 | Carer mood |  |  |  |  |  |  |  |  |  |  |  |
| Clark 2003 | Depression_GDS |  |  |  |  |  |  |  |  |  |  |  |
| Clark 2003 | Functional status_BI |  |  |  |  |  |  |  |  |  |  |  |
| Clark 2003 | Quality of Life_SF-36 |  |  |  |  |  |  |  |  |  |  |  |
| Clark 2003 | Self-efficacy_Mastery scale |  |  |  |  |  |  |  |  |  |  |  |
| Duncan 2020 | Cognition_Telephone MoCA |  |  |  |  |  |  |  |  |  |  |  |
| Duncan 2020 | Depression_PHQ-2 |  |  |  |  |  |  |  |  |  |  |  |
| Duncan 2020 | Falls |  |  |  |  |  |  |  |  |  |  |  |
| Duncan 2020 | Fatigue_PR0MIS Fatigue |  |  |  |  |  |  |  |  |  |  |  |
| Duncan 2020 | Functional status_MRS |  |  |  |  |  |  |  |  |  |  |  |
| Duncan 2020 | QOL_Likets scale |  |  |  |  |  |  |  |  |  |  |  |
| Duncan 2020 | Satisfaction with stroke care_CAHPS |  |  |  |  |  |  |  |  |  |  |  |
| Feng 2021 | Anxiety_SAS |  |  |  |  |  |  |  |  |  |  |  |
| Feng 2021 | Depression_SDS |  |  |  |  |  |  |  |  |  |  |  |
| Feng 2021 | Functional status_MBI |  |  |  |  |  |  |  |  |  |  |  |
| Feng 2021 | Self-efficacy_GSES |  |  |  |  |  |  |  |  |  |  |  |
| Geng 2019 | Functional status_MBI |  |  |  |  |  |  |  |  |  |  |  |
| Geng 2019 | QOL_SF-36 |  |  |  |  |  |  |  |  |  |  |  |
| Li 2020 | Functional status_MBI |  |  |  |  |  |  |  |  |  |  |  |
| Li 2020 | Self-efficacy_GSES |  |  |  |  |  |  |  |  |  |  |  |
| Lincoln 2003 | Caregiver Mood_GHQ-12 |  |  |  |  |  |  |  |  |  |  |  |
| Lincoln 2003 | Carer Burden_CSI |  |  |  |  |  |  |  |  |  |  |  |
| Lincoln 2003 | Depression_GHQ-12 |  |  |  |  |  |  |  |  |  |  |  |
| Lincoln 2003 | Functional status_BI |  |  |  |  |  |  |  |  |  |  |  |
| Lincoln 2003 | Satisfaction with stroke care_Likert |  |  |  |  |  |  |  |  |  |  |  |
| Liu 2018 | QOL_SF-36 |  |  |  |  |  |  |  |  |  |  |  |
| Lo 2018 | Self efficacy_SSEQ |  |  |  |  |  |  |  |  |  |  |  |
| Mayo 2008 | FS­_BI |  |  |  |  |  |  |  |  |  |  |  |
| Mayo 2008 | QOL­_EQ5D |  |  |  |  |  |  |  |  |  |  |  |
| Mayo 2008 | QOL­­_GDS |  |  |  |  |  |  |  |  |  |  |  |
| Mayo 2008 | QOL_PBSI |  |  |  |  |  |  |  |  |  |  |  |
| Mayo 2008 | QOL_SF-36 |  |  |  |  |  |  |  |  |  |  |  |
| Mayo 2008 | Readmission |  |  |  |  |  |  |  |  |  |  |  |
| Mayo 2008 | Use of HC services_GP |  |  |  |  |  |  |  |  |  |  |  |
| Mayo 2008 | Use of HC services_Specialised OPD |  |  |  |  |  |  |  |  |  |  |  |
| Mayo 2008 | ED |  |  |  |  |  |  |  |  |  |  |  |
| Reeves 2019 | Anxiety_Neuroqol anxiety |  |  |  |  |  |  |  |  |  |  |  |
| Reeves 2019 | Depression_PHQ-9 |  |  |  |  |  |  |  |  |  |  |  |
| Reeves 2019 | Functional status_simplified MRS |  |  |  |  |  |  |  |  |  |  |  |
| Reeves 2019 | QOL_Promis 10 |  |  |  |  |  |  |  |  |  |  |  |
| Reeves 2019 | Readmission |  |  |  |  |  |  |  |  |  |  |  |
| Reeves 2019 | Self-efficacy_PAM |  |  |  |  |  |  |  |  |  |  |  |
| Rodgers 1998 | QOL­_SF-36 |  |  |  |  |  |  |  |  |  |  |  |
| Rodgers 1999 | Anxiety_HADS-A |  |  |  |  |  |  |  |  |  |  |  |
| Rodgers 1999 | Depression_GHQ |  |  |  |  |  |  |  |  |  |  |  |
| Rodgers 1999 | Depression_HADS-D |  |  |  |  |  |  |  |  |  |  |  |
| Rodgers 1999 | FS_OHS |  |  |  |  |  |  |  |  |  |  |  |
| Rodgers 1999 | QOL_SF-36 |  |  |  |  |  |  |  |  |  |  |  |
| Rodgers 1999 | Satisfaction with stroke care |  |  |  |  |  |  |  |  |  |  |  |
| Watkins 2007 | Depression_GHQ-28 |  |  |  |  |  |  |  |  |  |  |  |
| Watkins 2007 | Depression_Yale |  |  |  |  |  |  |  |  |  |  |  |
| Watkins 2007 | Functional status_BI |  |  |  |  |  |  |  |  |  |  |  |
| Watkins 2007 | Self-efficacy_SEQ |  |  |  |  |  |  |  |  |  |  |  |
| Wong 2015 | ED |  |  |  |  |  |  |  |  |  |  |  |
| Wong 2015 | Depression_CESD-HK |  |  |  |  |  |  |  |  |  |  |  |
| Wong 2015 | Fuctional status_MBI |  |  |  |  |  |  |  |  |  |  |  |
| Wong 2015 | PSQ-HK |  |  |  |  |  |  |  |  |  |  |  |
| Wong 2015 | QOL_SF-36 |  |  |  |  |  |  |  |  |  |  |  |
| Wong 2015 | QOL_WHO-QOL-SRPB |  |  |  |  |  |  |  |  |  |  |  |
| Wong 2015 | Readmission |  |  |  |  |  |  |  |  |  |  |  |

**Table 7: GRADE summary of findings: Functional Status**

| **Summary of findings:** | | | | | | |
| --- | --- | --- | --- | --- | --- | --- |
| **Support intervention compared to usual care for stroke patients at transitions from hospital to home** | | | | | | |
| **Patient or population:** stroke patients at transitions from hospital to home  **Setting:**  **Intervention:** support intervention  **Comparison:** usual care | | | | | | |
| Outcomes | **Anticipated absolute effects^*^** (95% CI) | | Relative effect (95% CI) | № of participants (studies) | Certainty of the evidence (GRADE) | Comments |
|  | **Risk with usual care** | **Risk with support intervention** |  |  |  |  |
| Functional status assessed with: Barthel Index follow-up: range 7 days to 3 months |  | MD **7.87 points higher** (3.93 higher to 11.81 higher) | - | 620 (5 RCTs) | ⨁◯◯◯ Very low^a,b,c,d,e^ |  |
| Functional status assessed with: Barthel Index follow-up: range 6 months to 12 months |  | MD **2.91 points higher** (0.03 higher to 5.8 higher) | - | 1827 (6 RCTs) | ⨁◯◯◯ Very low^f,g,h,i^ |  |
| Depression assessed with: Varied follow-up: range 6 months to 12 months | - | SMD **0.17 SD lower** (0.29 lower to 0.05 lower) | - | 1041 (4 RCTs) | ⨁⨁◯◯ Low^j,k^ |  |
| Anxiety assessed with: HADS-A follow-up: mean 6 months |  | MD **1.7 points lower** (1.84 lower to 0.52 lower) | - | 601 (2 RCTs) | ⨁◯◯◯ Very low^l,m,n^ |  |
| Quality of life (QOL) assessed with: SF-36 - PCS follow-up: range 4 weeks to 3 months |  | MD **1.3 points higher** (0.85 higher to 1.76 higher) | - | 331 (3 RCTs) | ⨁◯◯◯ Very low^o,p,q^ |  |
| ***The risk in the intervention group** (and its 95% confidence interval) is based on the assumed risk in the comparison group and the **relative effect** of the intervention (and its 95% CI).  **CI:** confidence interval; **MD:** mean difference; **SMD:** standardised mean difference | | | | | | |
| **GRADE Working Group grades of evidence** **High certainty:** we are very confident that the true effect lies close to that of the estimate of the effect. **Moderate certainty:** we are moderately confident in the effect estimate: the true effect is likely to be close to the estimate of the effect, but there is a possibility that it is substantially different. **Low certainty:** our confidence in the effect estimate is limited: the true effect may be substantially different from the estimate of the effect. **Very low certainty:** we have very little confidence in the effect estimate: the true effect is likely to be substantially different from the estimate of effect. | | | | | | |

#### Explanations

a. Graded down by 1 for ROB: (1)incomplete outcome data: high risk of bias for two studies which contribute almost 1/3 to the overall body of evidence. 2) Blinding was not possible in these trials leading to some concerns, but in 3/5 trials intention to treat was not described.

b. Inconsistency: downgraded by 1 based on high percentage of I squared (77%); variation in point estimates; and slight concern by Mayo 2008 (lack of detail in intervention, would need to do sensitivity analysis)

c. Indirectness: not downgraded - slight concern by Mayo 2008 (lack of detail in intervention, would need to do sensitivity analysis)

d. Downgraded by 1 for imprecision due to low study size (<1000) and lower limit of confidence interval crosses threshold.

e. As the number of studies is too low to perform tests for publication bias we cannot be sure we have obtained all the relevant studies (<10)

f. Graded down by 1 for ROB: (1) incomplete outcome data: high risk of bias for two studies which contribute 1/3 to the overall body of evidence. 2) Blinding to intervention was not possible in these trials leading to some concerns, but in 3/6 trials intention to treat was not described.

g. Inconsistency: downgraded by 2 based on high percentage of I squared (84%); variation in point estimates; limited overlap in confidence intervals, and concern by Boter 2004 (not estimatable), baseline functional differences between participants in Lincoln 2003, and Mayo 2008 lack of detail in intervention (would need to do sensitivity analysis)

h. Downgraded by 1 for imprecision due to confidence interval cross threshold for 3 studies.

i. As the number of studies is too low to perform tests for publication bias we cannot be sure we have obtained all the relevant studies (<10)

j. Graded down by 1 for ROB: (1)incomplete outcome data 2) Blinding was not possible in these trials leading to some concerns, 3) all trials had some concerns D4

k. As the number of studies is too low to perform tests for publication bias we cannot be sure we have obtained all the relevant studies (<10)

l. Graded down by 1 for ROB: D2 2 studies with some concerns or high ROB, D4 all studies with high ROB and concerns or high ROB for all studies in selection of reported results. Missing outcome data in 1 study

m. Downgraded by 1 for imprecision due to low study size (<1000), very wide confidence interval in one study

n. As the number of studies is too low to perform tests for publication bias we cannot be sure we have obtained all the relevant studies (<10)

o. Graded down by 1 for ROB: D1 2 studies with some concerns, D2 1 studies with high ROB, D4 all studies with high ROB, and concerns or high ROB for all studies in selection of reported results.

p. Downgraded by 1 for imprecision due to low study size (<200 in each group)

q. As the number of studies is too low to perform tests for publication bias we cannot be sure we have obtained all the relevant studies (<10)

Analysis 1.1 Up to 3-months


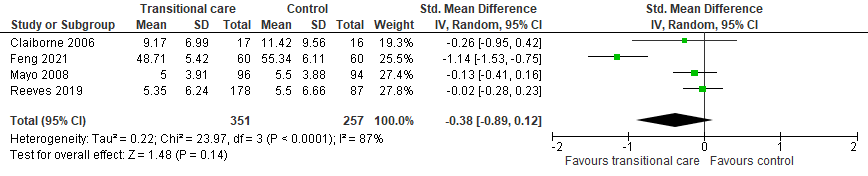


Analysis 1.2 Between 6-12 months


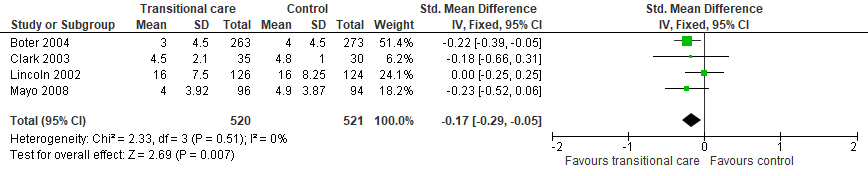


**S1. Forest Plot of Depression Outcome: Transitional care intervention vs. Control**

Analysis 2.1 Up to 3-months

**
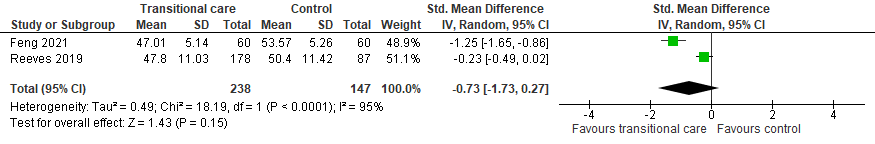
**

Analysis 2.2 Between 6-12 months


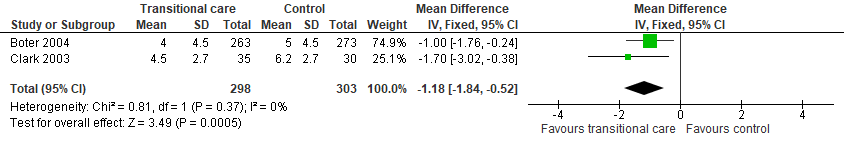


**S2. Forest Plot of Anxiety Outcome: Transitional care intervention vs. Control**

Analysis 3.1 Physical component score (PCS): Up to 3-months


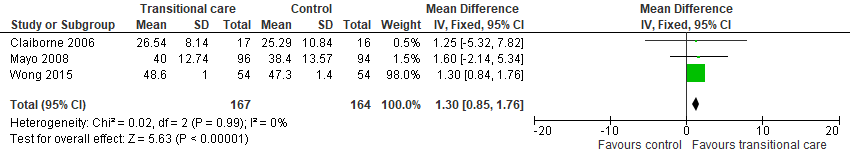


Analysis 3.2 Mental component score (MCS) Up to 3-months


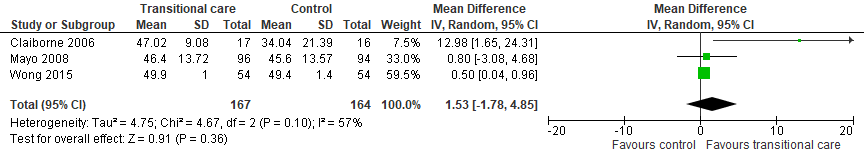


Analysis 3.3 Physical component score (PCS): Between 6-12 months


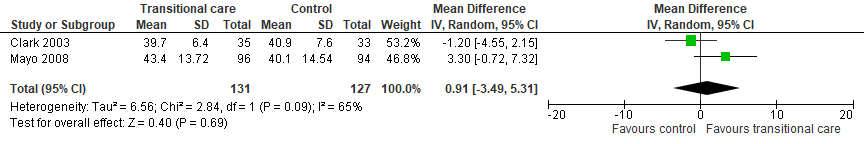


Analysis 3.4 Mental component score (MCS) between 6-12 months


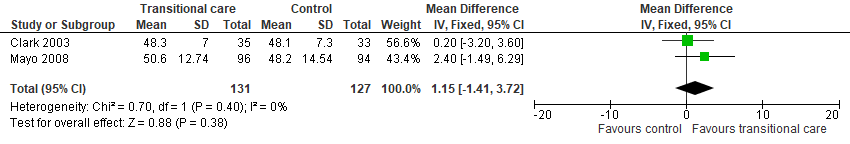


**S3. Forest Plot of Quality of Life Outcome: Transitional care intervention vs. Control**

Analysis 4.1 Up to 3-months


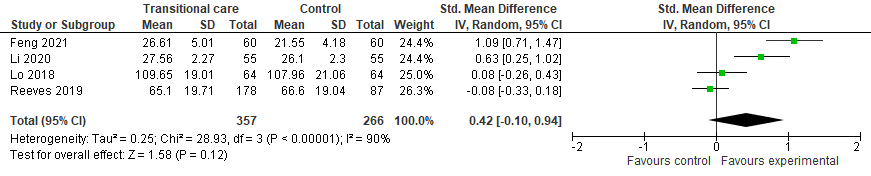


Analysis 4.2 Between 6-12 months


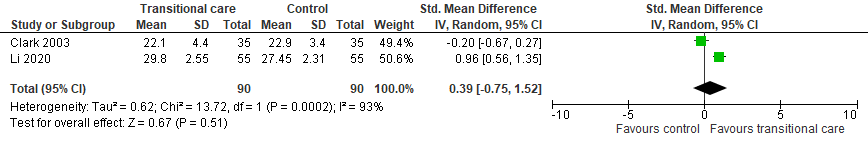


**S4. Forest Plot of Self-efficacy Outcome: Transitional care intervention vs. Control**

Analysis 5.1 Hospital readmission at end of follow-up


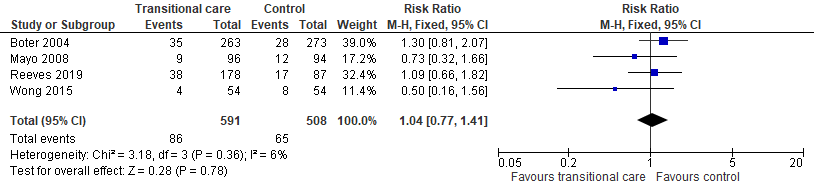


Analysis 5.2 ED visits at end of follow-up

**
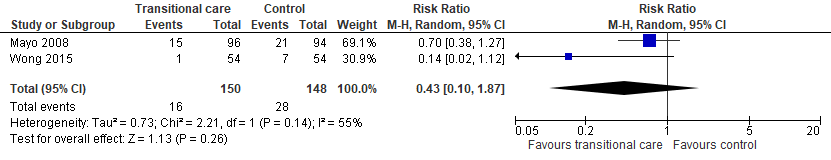
**

Analysis 5.3 GP visits end of follow-up


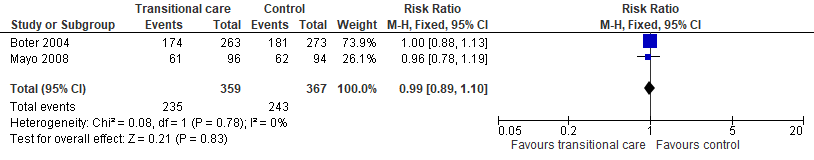


**S5. Forest Plot of Healthcare Utilisation Outcomes: Transitional care intervention vs. Control**

Analysis 6.1 Caregiver strain between 6-12 months


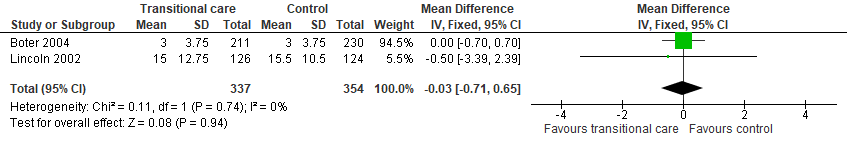


**S6. Forest Plot of Caregiver Strain Outcome: Transitional care intervention vs. Control.**

**Sensitivity analysis I**

**Functional Status**

1. **Risk Of Bias (ROB)**

All studies had *Some concerns* or overall *High ROB* (Figure I & 2) therefore sensitivity analysis not appropriate.


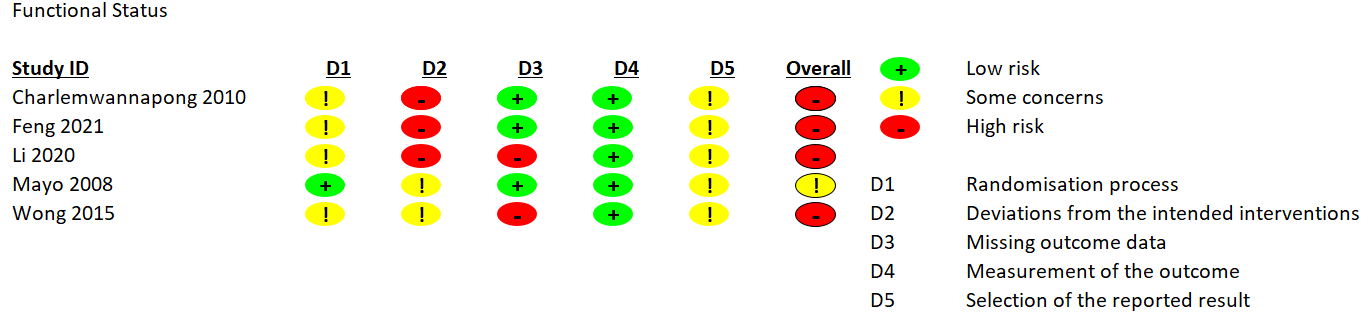


Figure I. Risk of Bias for Outcome: Functional status (Up to 3 months)


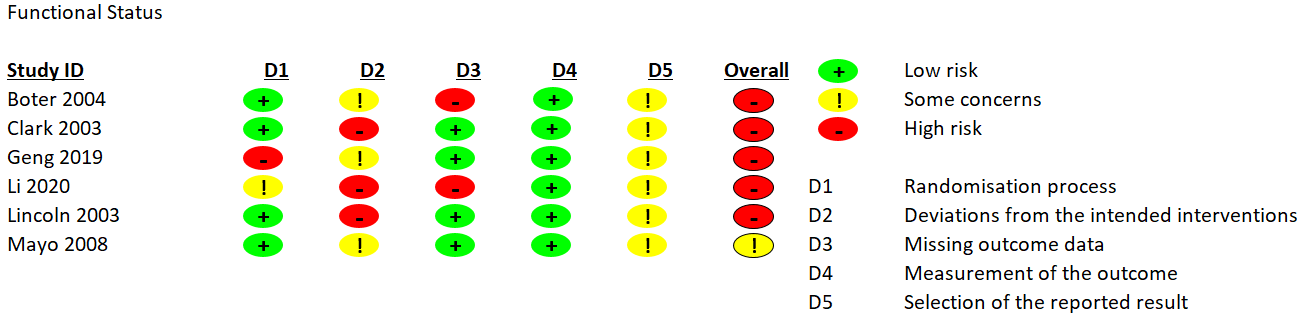


Figure 2. Risk of Bias for Outcome: Functional status (Between 6-12 months)

1. **Barthel Index (BI) outliers**

BI scores were measured as continuous variables ranging from 0 to 100, or using a short version of the Barthel Index with scores ranging from 0 to 20. These scores are comparable. BI was also measured using a Likert score^18^ and by omitting one domain as participants did not have stairs^2^. Sensitivity analysis was carried out to remove BI outliers (Figure I).


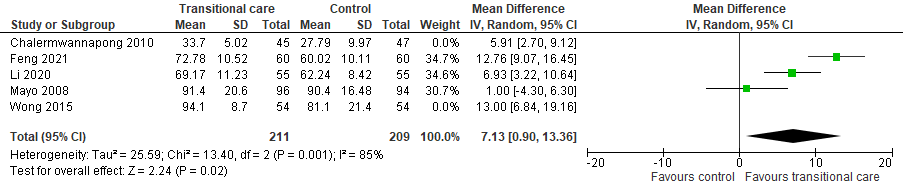


Figure I. Sensitivity analysis Barthel Index outliers

1. **Selection Bias**

Sensitivity analysis was carried out to remove studies with selection bias^8^(Figure I).


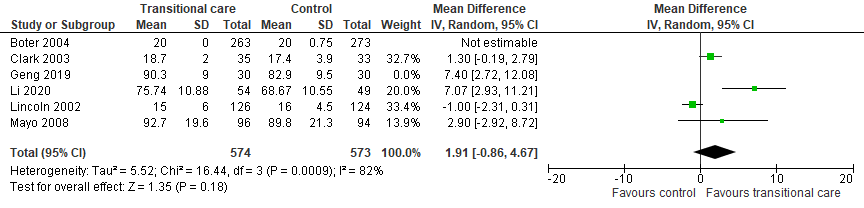


Figure I. Sensitivity analysis: Selection Bias

1. **Quasi randomisation**

Sensitivity analysis was carried out to remove quasi-randomised studies^8^(Figure I).


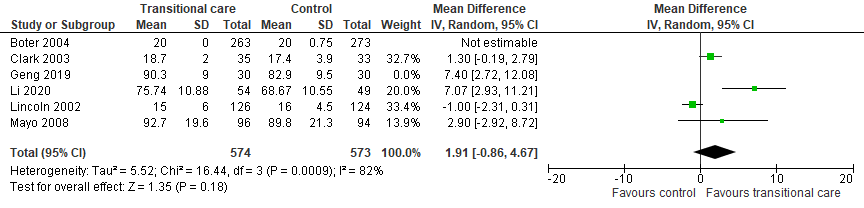


Figure I. Sensitivity analysis: Quasi-randomisation

1. **Missing Outcomes Bias**

Sensitivity analysis was carried out to address missing outcomes bias up to 3 months ^9, 18^ (Figure I) and between 6-12 months ^1, 9^ (Figure 2).

**
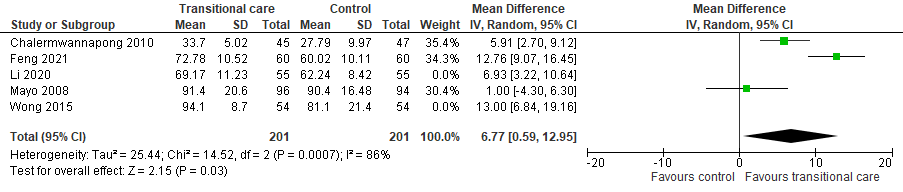
**

Figure I. Missing outcome bias (Up to 3 months)

**
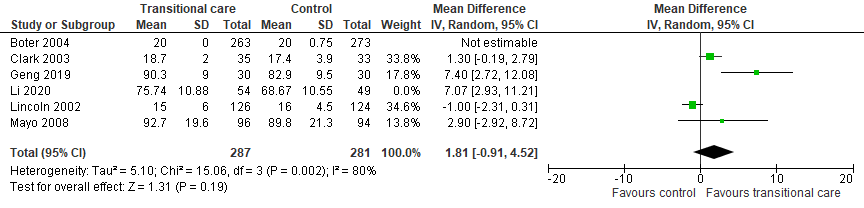
**

Figure 2. Missing outcome bias (Between 6-12 months)

1. **Entering assumed values**

Sensitivity analysis was carried out to address entering assumed values up to 3 months^13^ (Figure I) and between 6-12 months^1, 10, 13^ (Figure 2)

**
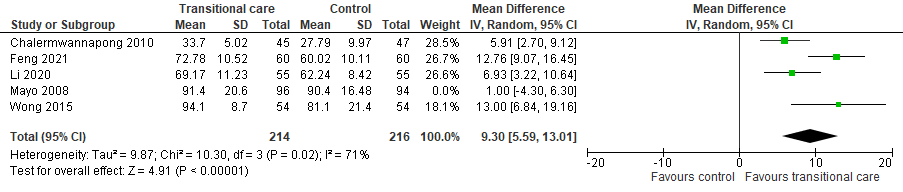
**

Figure I. Entering assumed values (Up to 3 months)

**
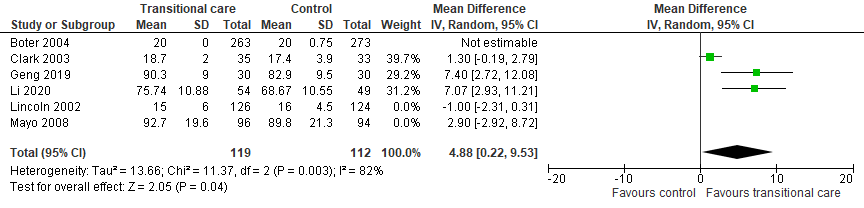
**

Figure 2. Entering assumed values (Between 6-12 months)

**Sensitivity analysis 2**

**Depression**

1. **Risk Of Bias (ROB)**

All studies had *Some concerns* or overall *High ROB* (Figure I & 2) therefore sensitivity analysis not appropriate.


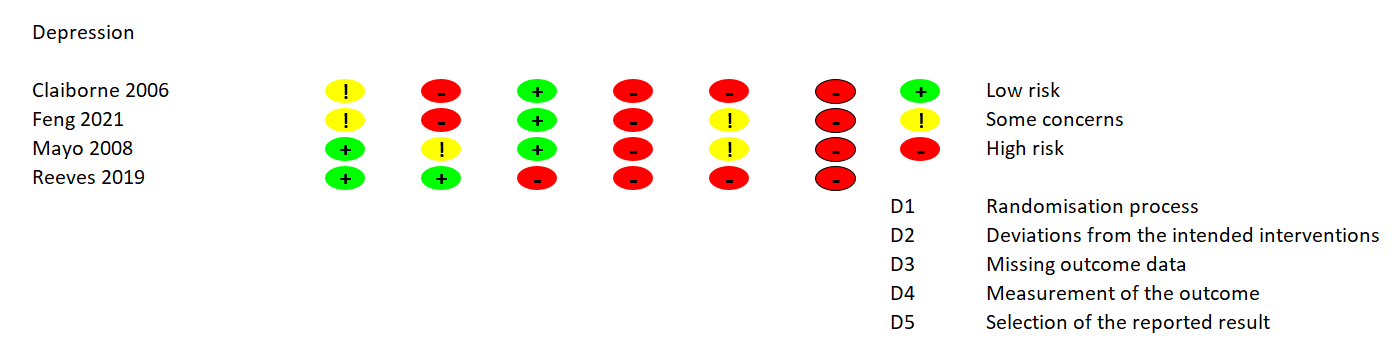


Figure I. Risk of Bias for Outcome: Depression (Up to 3 months)


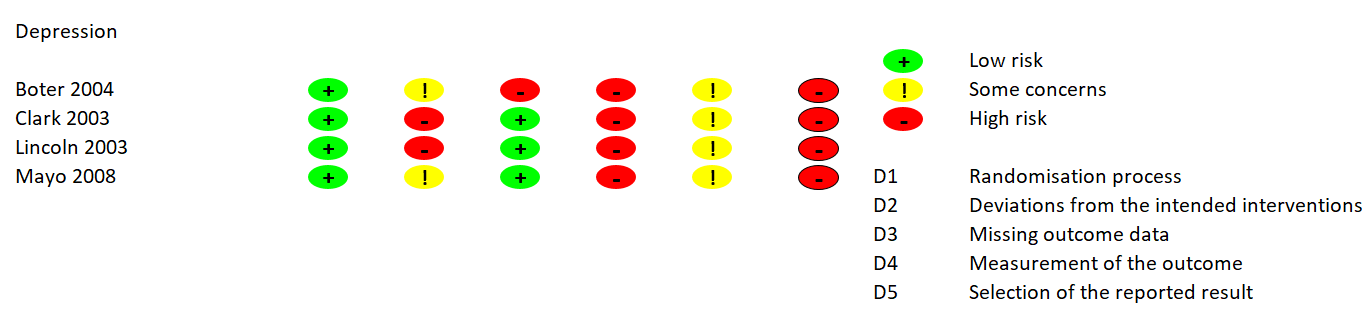


Figure 2. Risk of Bias for Outcome: Depression (Between 6-12 months)

1. **Missing Outcomes Bias**

Sensitivity analysis was carried out to address missing outcomes bias up to 3 months^14^ (Figure I) and between 6-12 months^1^ (Figure 2).

**
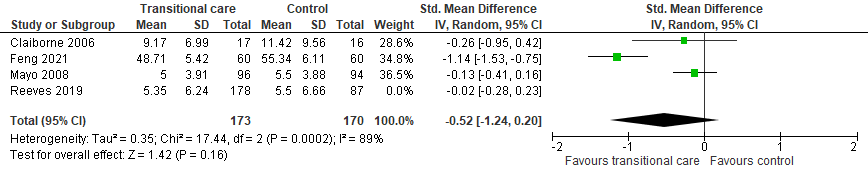
**

Figure I. Missing outcome bias (Up to 3 months)

**
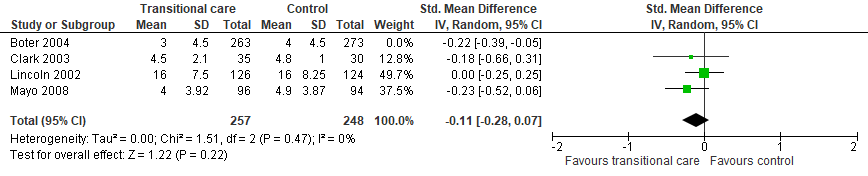
**

Figure 2. Missing outcome bias (Between 6-12 months)

1. **Entering assumed values**

Sensitivity analysis was carried out to address entering assumed values up to 3 months^14^ (Figure I) and between 6-12 months^1, 10, 13^ (Figure 2)

**
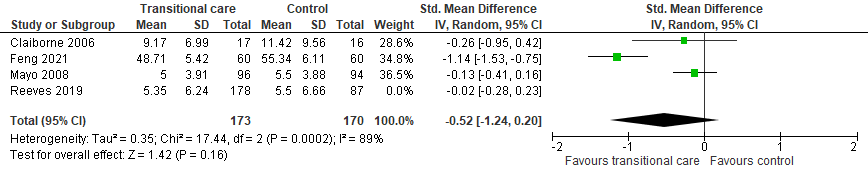
**

Figure I. Entering assumed values (Up to 3 months)

**
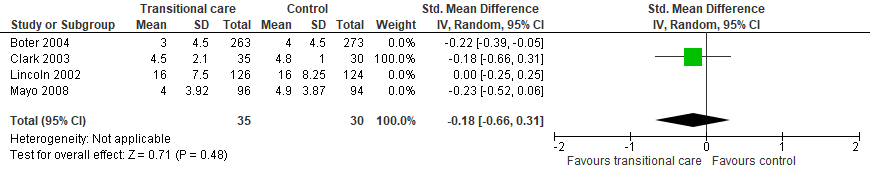
**

Figure 2. Entering assumed values (Between 6-12 months)

**Sensitivity analysis 3**

**Anxiety**

1. **Risk of Bias (ROB)**

All studies had *Some concerns* or overall *High ROB* (Figure I & 2) therefore sensitivity analysis not appropriate.


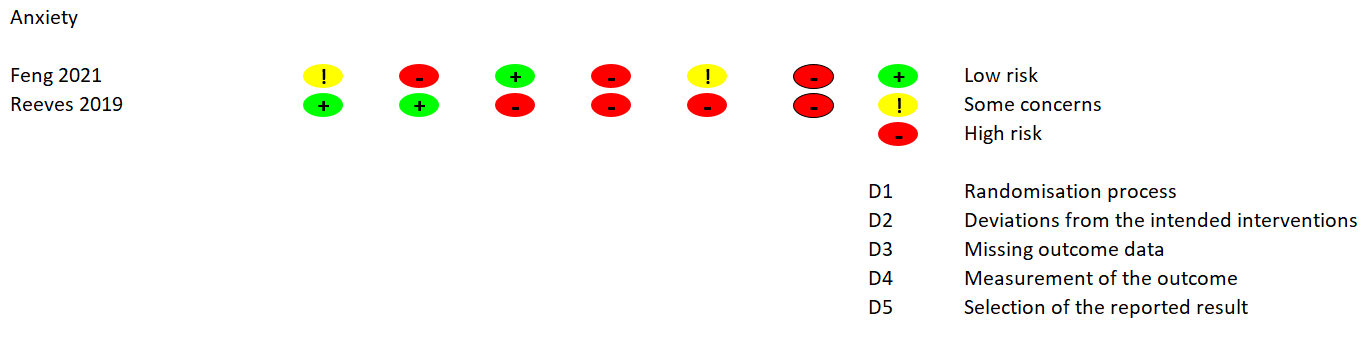


Figure I. Risk of Bias for Outcome: Anxiety (Up to 3 months)


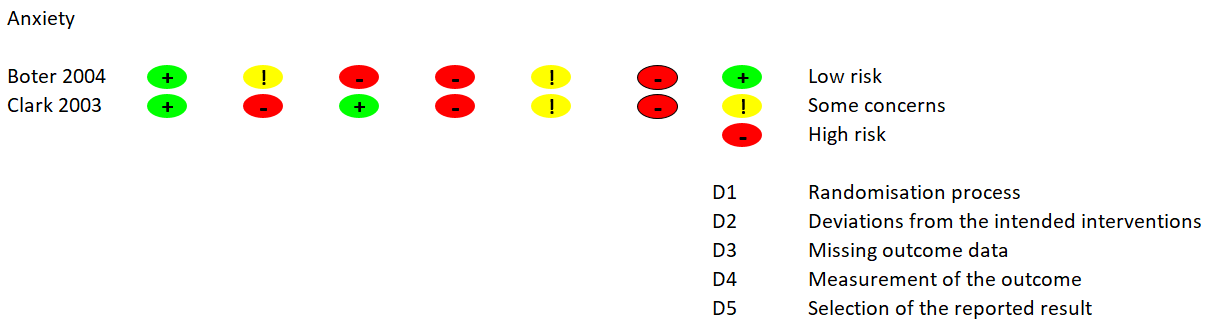


Figure 2. Risk of Bias for Outcome: Anxiety (Between 6-12 months)

1. **Missing Outcomes Bias**

Sensitivity analysis was carried out to address missing outcomes bias up to 3 months^7^ (Figure I) and between 6-12 months^1^ (Figure 2).

**
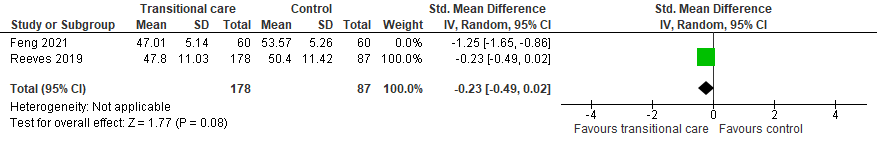
**

Figure I. Missing outcome bias (Up to 3 months)

**
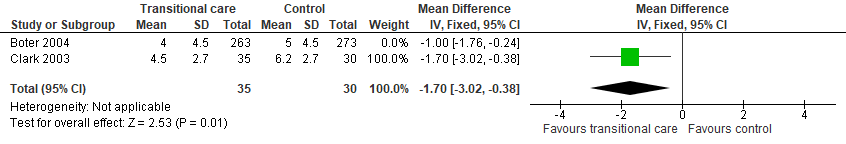
**

Figure 2. Missing outcome bias (Between 6-12 months)

1. **Entering assumed values**

Sensitivity analysis was carried out to address entering assumed values up to 3 months^14^ (Figure I) and between 6-12 months^1^ (Figure 2)

**
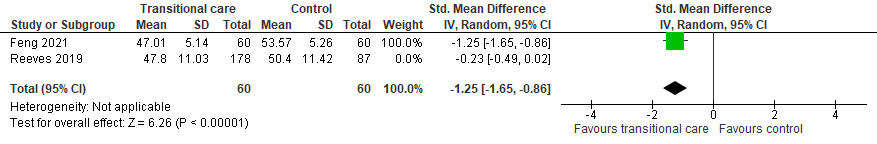
**

Figure I. Entering assumed values (Up to 3 months)

**
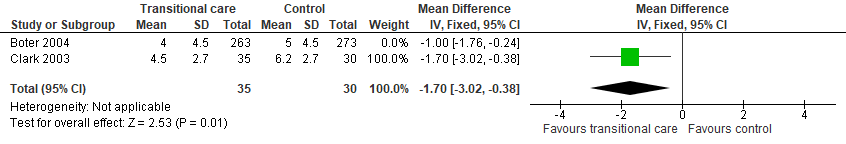
**

Figure 2. Entering assumed values (Between 6-12 months)

**Sensitivity analysis** 4

**Quality of life (QOL)**

1. **Risk of Bias (ROB)**

All studies had *Some concerns* or overall *High ROB* (Figure I & 2) therefore sensitivity analysis not appropriate.


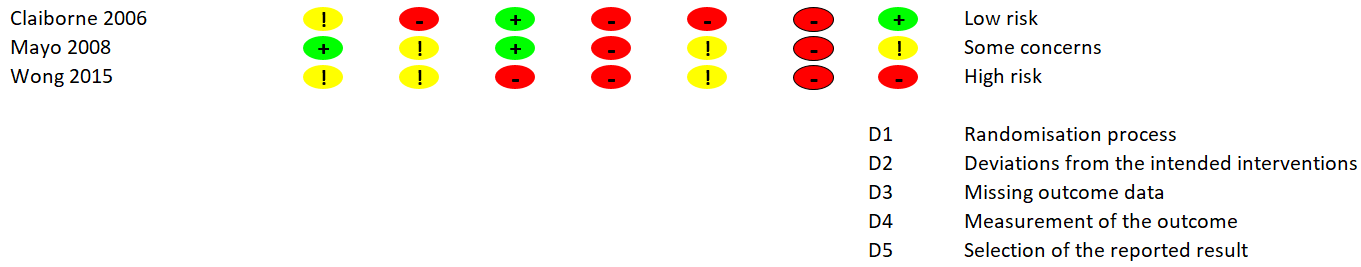


Figure I. Risk of Bias for Outcome: Quality of life (Up to 3 months)


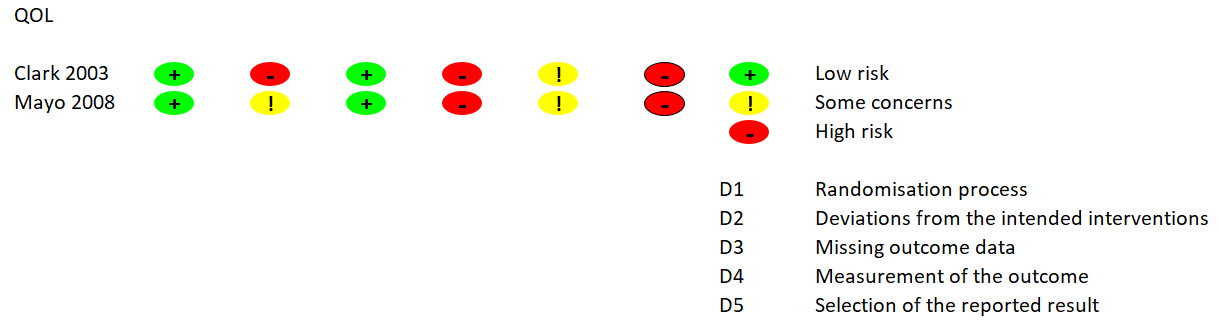


Figure 2. Risk of Bias for Outcome: Quality of life (Between 6-12 months)

1. **Missing Outcomes Bias**

Sensitivity analysis was carried out to address missing outcomes bias up to 3 months^18^ (Figure Ia Physical component score (PCS) & Figure Ib Mental component score (MCS)). Not applicable between 6-12 months.

**
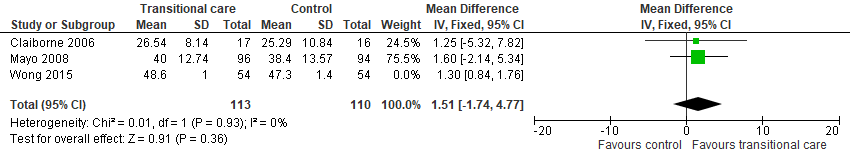
**Figure Ia. Missing outcome bias PCS (Up to 3 months)

**
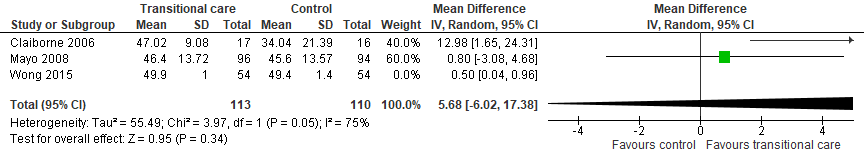
**

Figure Ib. Missing outcome bias MCS (Up to 3 months)

1. **Entering assumed values**

Sensitivity analysis was carried out to address entering assumed values up to 3 months^13^ (Figure Ia PCS & Figure Ib MCS) and between 6-12 months^13^ (Figure 2a PCS & Figure 2b MCS)

**
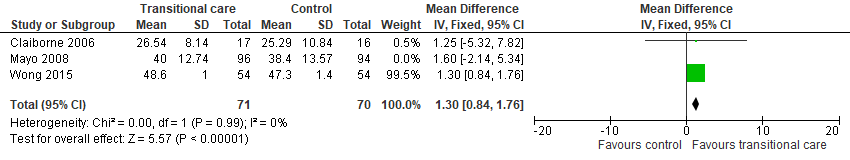
** Figure Ia. Entering assumed values PCS (Up to 3 months)

**
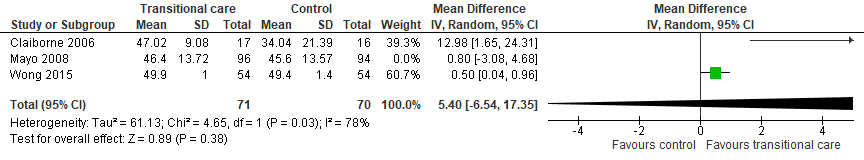
**

Figure Ib. Entering assumed values MCS (Up to 3 months)

**
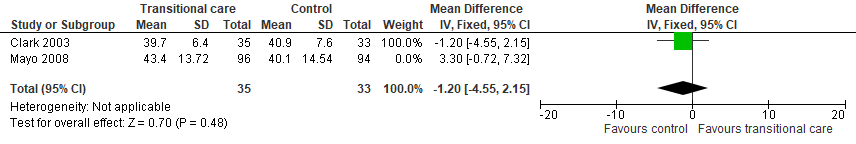
**

Figure 2a. Entering assumed values PCS (Between 6-12 months)

**
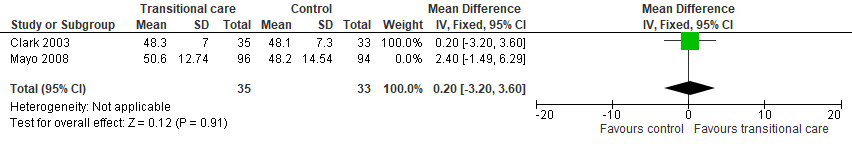
**

Figure 2b. Entering assumed values MCS (Between 6-12 months)

**Sensitivity analysis** 5

**Self-efficacy**

1. **Risk of Bias (ROB)**

All studies had *Some concerns* or overall *High ROB* (Figure I & 2) therefore sensitivity analysis not appropriate.


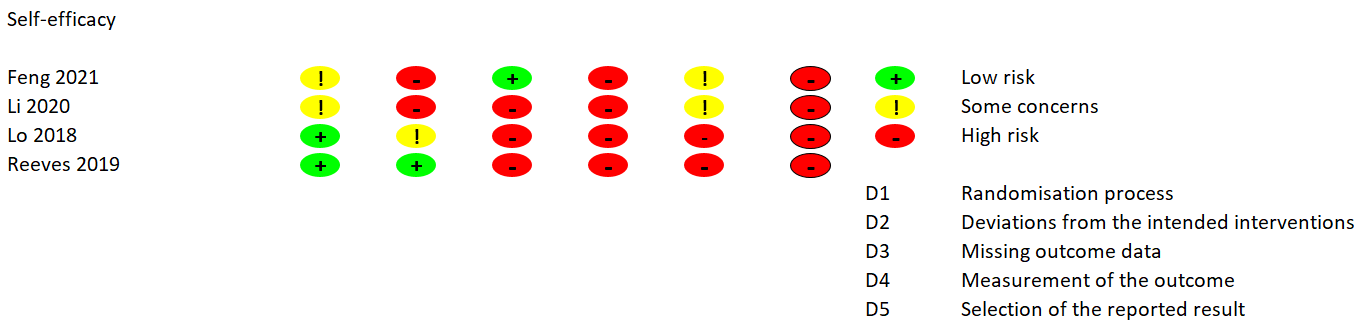


Figure I. Risk of Bias for Outcome: Self-efficacy (Up to 3 months)


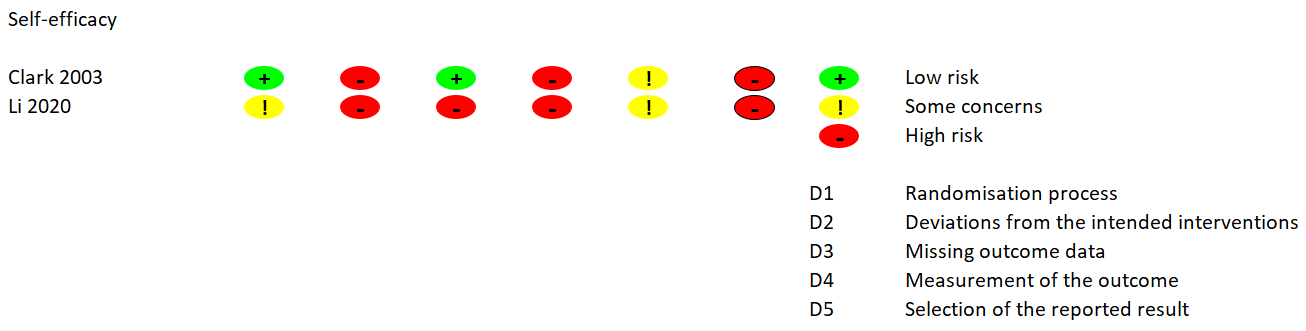


Figure 2. Risk of Bias for Outcome: Self-efficacy (Between 6-12 months)

1. **Missing Outcomes Bias**

Sensitivity analysis was carried out to address missing outcomes bias up to 3 months^9, 12, 14^ (Figure I) and between 6-12 months^9^ (Figure 2).

**
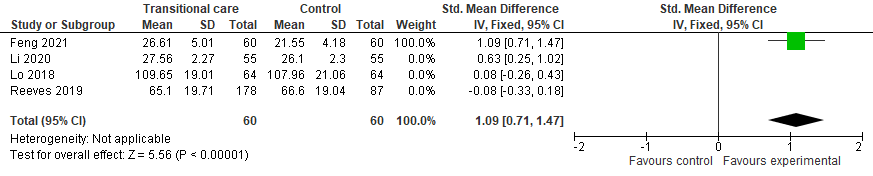
**

Figure I. Missing outcome bias (Up to 3 months)

**
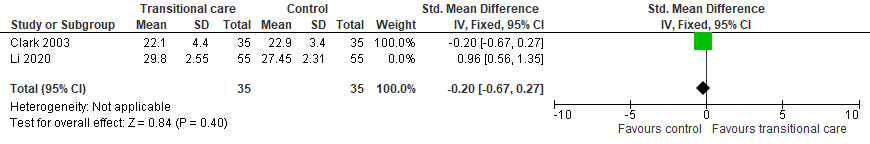
**

Figure 2. Missing outcome bias (Between 6-12 months)

1. **Entering assumed values**

Sensitivity analysis was carried out to address entering assumed values up to 3 months^14^ (Figure I). Not applicable between 6-12 months.

**
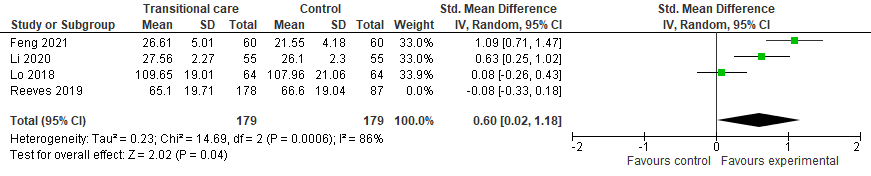
**

Figure I. Entering assumed values (Up to 3 months)

**Sensitivity analysis 6**

**Healthcare Utilisation**

1. **Risk of Bias (ROB)**

All studies had *Some concerns* or overall *High ROB* (Figure I) therefore sensitivity analysis not appropriate for Healthcare utilisation, Emergency Department (ED) visits and GP visits.


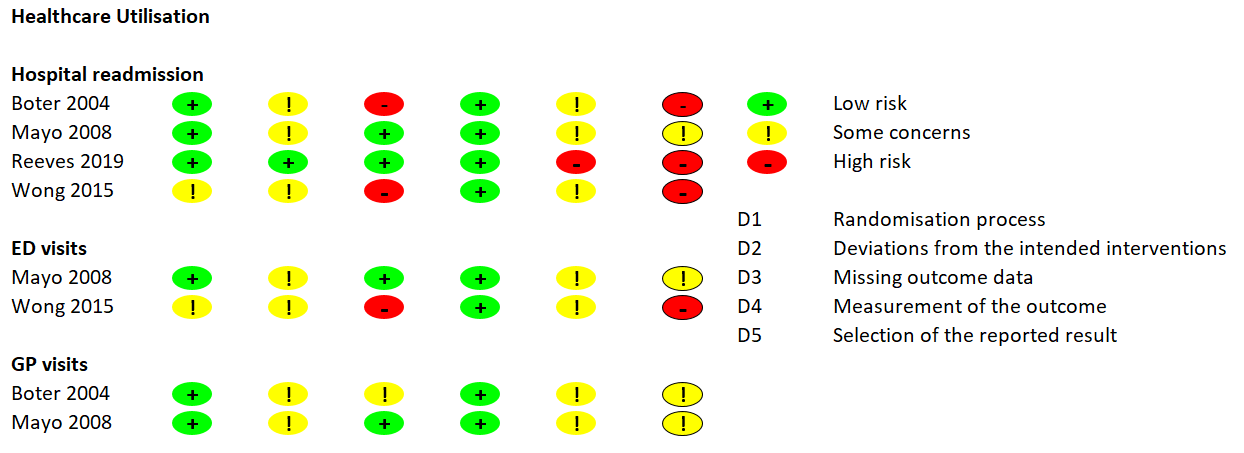


Figure I. Risk of Bias for Outcome: Healthcare Utilisation (End of follow-up)

1. **Missing Outcomes Bias**

Sensitivity analysis was carried out to address missing outcomes bias at end of follow up for hospital readmission^1, 18^ (Figure I) and ED Visits^18^(Figure 2). Not applicable for GP visits.

**
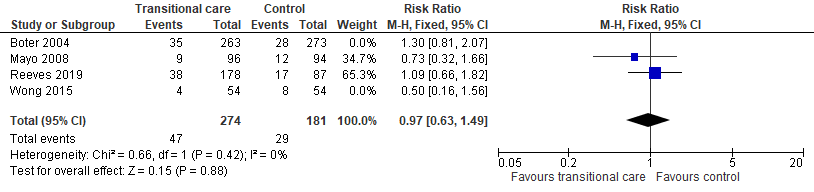
**

Figure I. Missing outcome bias for readmission (End of follow-up)

**
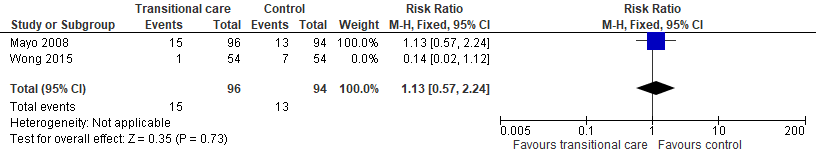
**

Figure 2. Missing outcome bias for ED visits (End of follow-up)

1. **Entering assumed values**

Sensitivity analysis (entering assumed values) for healthcare utilisation outcomes was not applicable

**Subgroup analysis:**

Subgroup analysis was carried out for functional status, depression, anxiety and self-efficacy, to identify variances based on:

1. Duration of intervention
2. Studies that recruited patient and caregiver/spouse
3. Theoretical underpinning
4. Studies that delivered a component specifically to the caregiver.

**I.Functional Status**


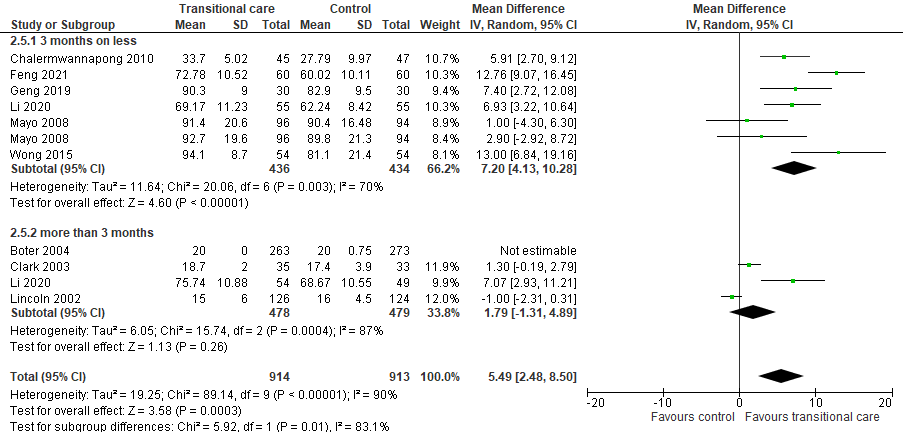


Figure 1. Duration of intervention: favours shorter intervention


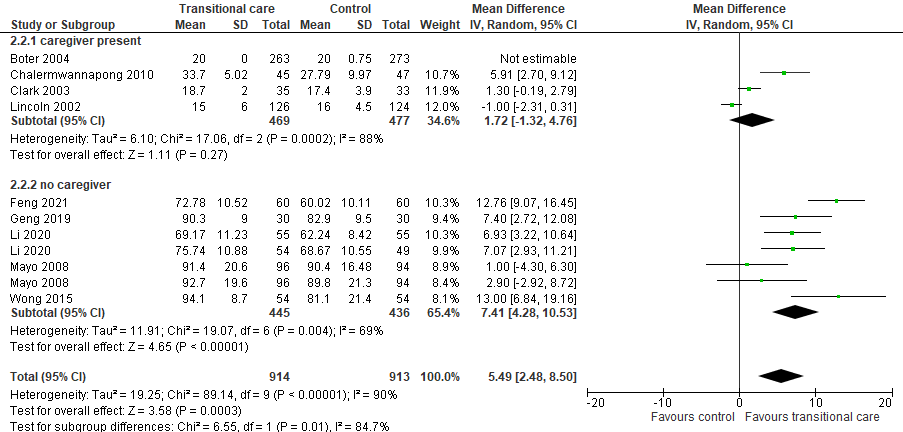
Figure 2. Caregiver recruited: Favours studies that recruited no caregiver


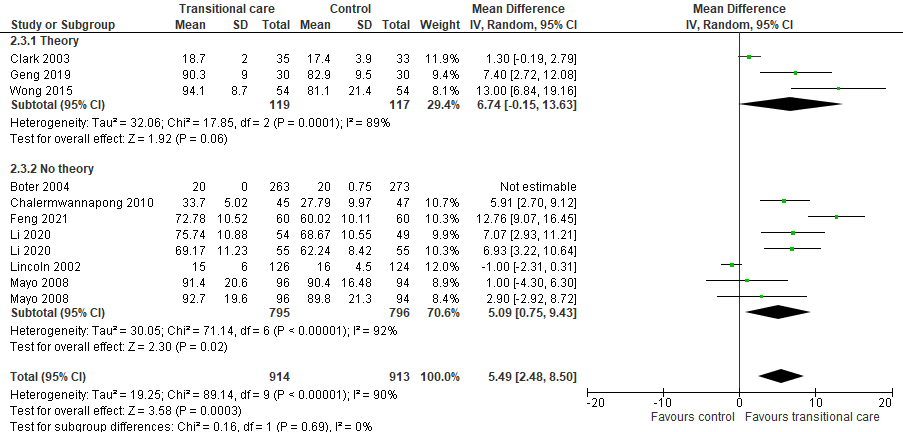


Figure 3. Theoretical underpinning: favours no theoretical underpinning

- Component specifically delivered to caregiver: not appropriate to analyse
- In all case Lincoln is outlier as people had poorer baseline function (more weakness in upper and lower limb)

**2. Depression**


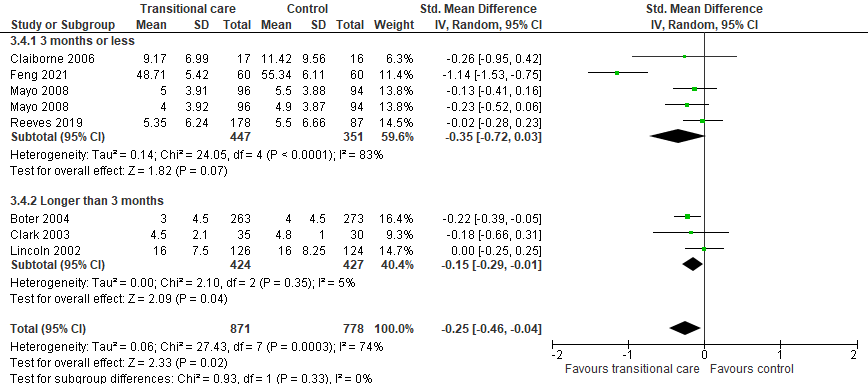


Figure I. Duration of intervention: favours longer intervention


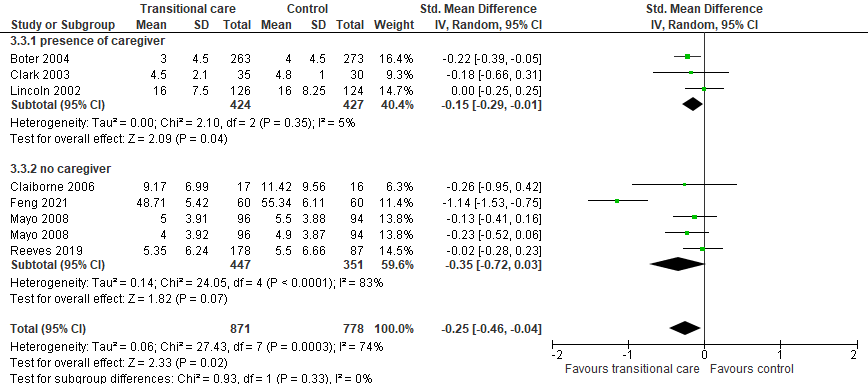


Figure 2. Caregiver recruited: Favours studies that recruited a caregiver


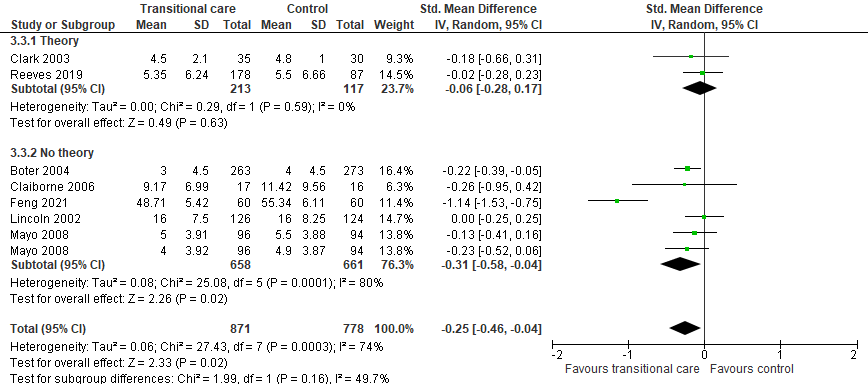


Figure 3. Theoretical underpinning: Favours no theoretical underpinning

**
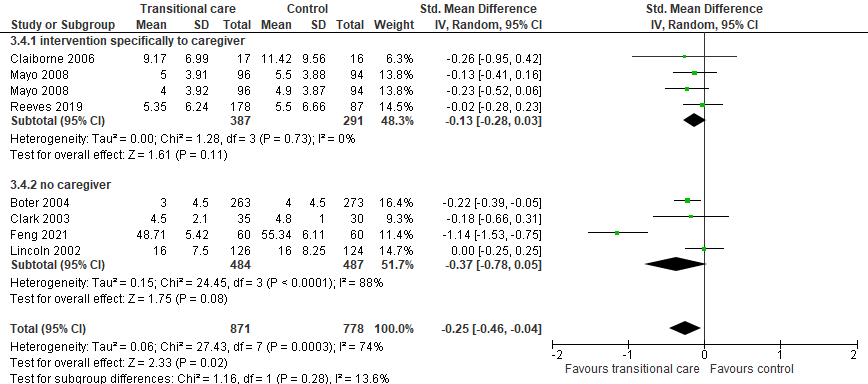
**

Figure 4. Component specifically delivered to caregiver: No effect

**3. Anxiety**


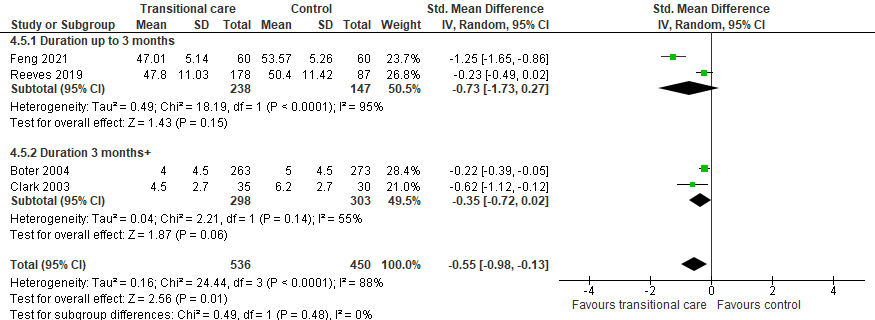


Figure I. Duration of intervention: favours longer duration


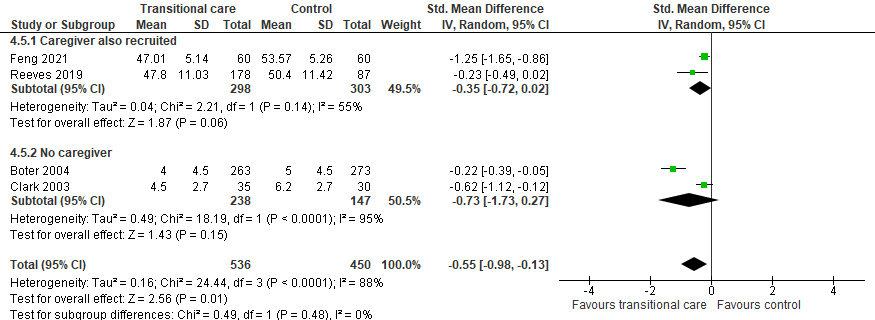


Figure 2. Caregiver recruited: No effect


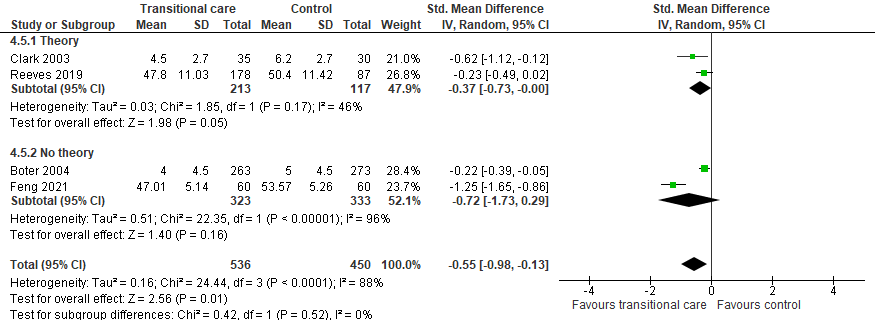


Figure 3. Theoretical underpinning: Favours theoretical underpinning


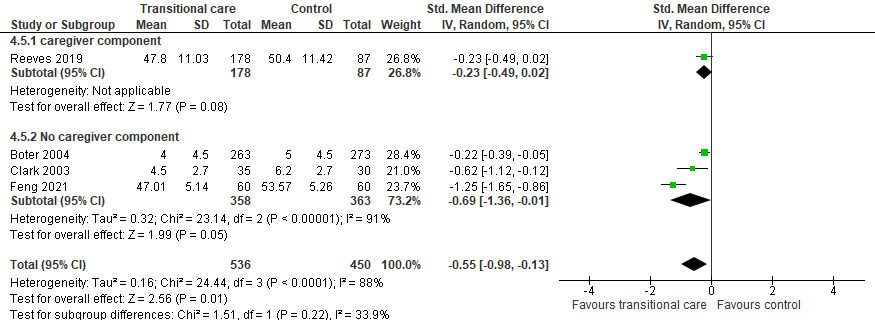


Figure 4. Component specifically delivered to caregiver: favours no caregiver component

4.**Self-efficacy**


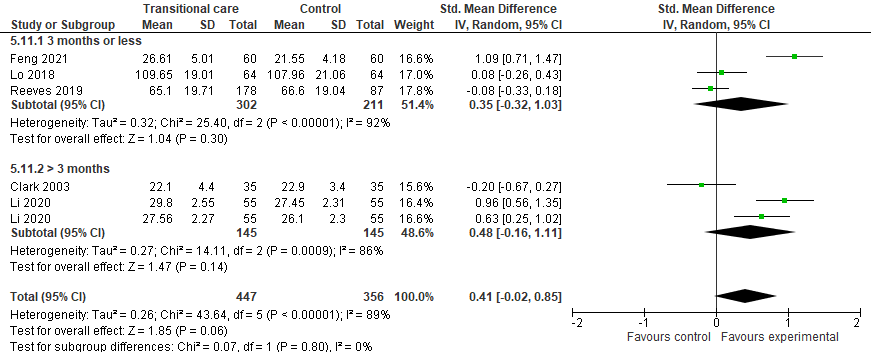


Figure I. Duration of intervention: no effect


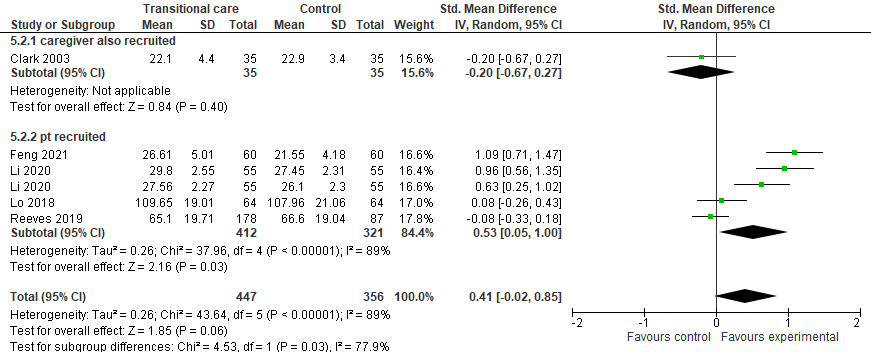


Figure 2. Caregiver recruited: Favours studies that recruited no caregiver


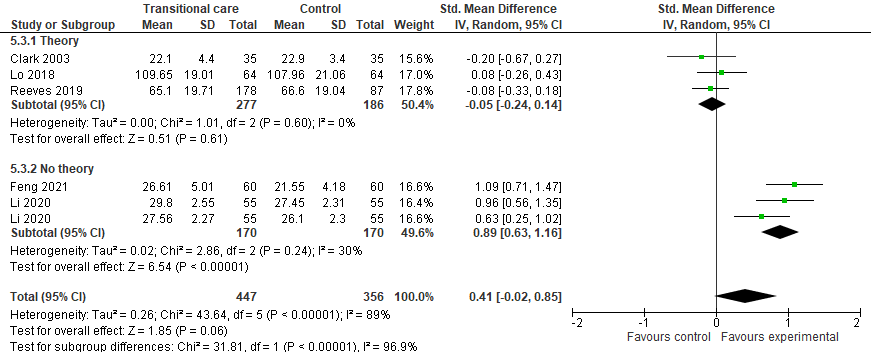


Figure 3. Theoretical underpinning: Favours no theoretical underpinning


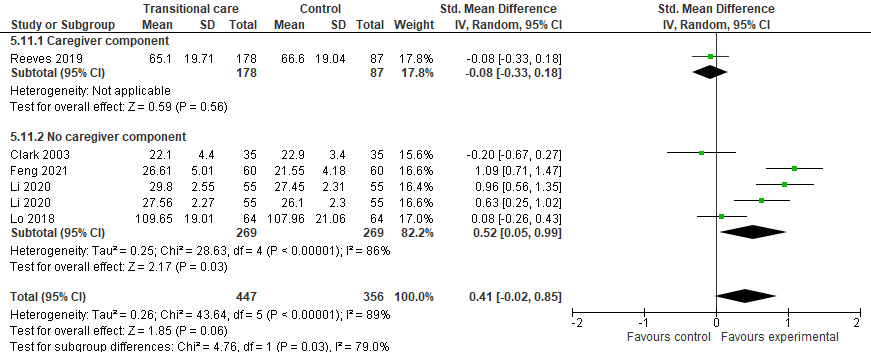


Figure 4. Component specifically delivered to caregiver: favours no caregiver component

**References (As per main document)**

1. Boter H. Multicenter randomized controlled trial of an outreach nursing support program for recently discharged stroke patients. *Stroke*. 2004;35:2867-2872

2. Chalermwannapong S, Panuthai S, Srisuphan W, Panya P, Ostwald SK. Effects of the transitional care program on functional ability and quality of life of stroke survivors. *CMU J. Nat. Sci*. 2010;9:49-66

3. Chen L, Chen Y, Chen X, Shen X, Wang Q, Sun C. Longitudinal study of effectiveness of a patient‐centered self‐management empowerment intervention during predischarge planning on stroke survivors. *J Worldviews on Evidence‐Based Nursing*. 2018;15:197-205

4. Claiborne N. Effectiveness of a care coordination model for stroke survivors: A randomized study. *Health & Social Work*. 2006;31:87-96

5. Clark MS, Rubenach S, Winsor A. A randomized controlled trial of an education and counselling intervention for families after stroke. *Clinical Rehabilitation*. 2003;17:703-712

6. Duncan PW, Bushnell CD, Jones SB, Psioda MA, Gesell SB, D'Agostino Jr RB, et al. Randomized pragmatic trial of stroke transitional care: The compass study. *Circulation: Cardiovascular Quality and Outcomes*. 2020;13:e006285

7. Feng W, Yu H, Wang J, Xia J. Application effect of the hospital-community integrated service model in home rehabilitation of stroke in disabled elderly: A randomised trial. *Annals of Palliative Medicine*. 2021;10:4670-4677

8. Geng G, He W, Ding L, Klug D, Xiao Y. Impact of transitional care for discharged elderly stroke patients in china: An application of the integrated behavioral model. *Topics in stroke rehabilitation*. 2019;26:621-629

9. Li M, Gai H, Nie Y, Chen H, Ma M, Wang L. Application of transitional care in hemiplegia patients during recovery of ischemic stroke via cloud follow-up system. *International Journal of Science*. 2020;7:136-141

10. Lincoln NB, Francis VM, Lilley SA, Sharma JC, Summerfield M. Evaluation of a stroke family support organiser: A randomized controlled trial. *Stroke*. 2003;34:116-121

11. Liu L. Effects of transitional care on functional exercise: Compliance and health status of stroke patients. *ACTA MEDICA MEDITERRANEA*. 2018;34:959-965

12. Lo SH, Chang AM, Chau JP. Stroke self-management support improves survivors’ self-efficacy and outcome expectation of self-management behaviors. *Stroke*. 2018;49:758-760

13. Mayo NE, Nadeau L, Ahmed S, White C, Grad R, Huang A, et al. Bridging the gap: The effectiveness of teaming a stroke coordinator with patient's personal physician on the outcome of stroke. *Age and ageing*. 2008;37:32-38

14. Reeves MJ, Fritz MC, Woodward AT, Hughes AK, Coursaris CK, Swierenga SJ, et al. Michigan stroke transitions trial: A clinical trial to improve stroke transitions. *Circulation: Cardiovascular Quality and Outcomes*. 2019;12:e005493

15. Rodgers H, Atkinson C, Bond S, Suddes M, Dobson R, Curless R. Randomized controlled trial of a comprehensive stroke education program for patients and caregivers. *Stroke*. 1999;30:2585-2591

16. Watkins CL, Auton MF, Deans CF, Dickinson HA, Jack CI, Lightbody CE, et al. Motivational interviewing early after acute stroke: A randomized, controlled trial. *Stroke*. 2007;38:1004-1009

17. Watkins CL, Wathan JV, Leathley MJ, Auton MF, Deans CF, Dickinson HA, et al. The 12-month effects of early motivational interviewing after acute stroke: A randomized controlled trial. *Stroke*. 2011;42:1956-1961

18. Wong FKY, Yeung SM. Effects of a 4‐week transitional care programme for discharged stroke survivors in h ong k ong: A randomised controlled trial. *Health and social care in the community*. 2015;23:619-631
